# Safety and tolerability of erenumab in individuals with episodic or chronic migraine across age groups: a pooled analysis of placebo-controlled trials

**DOI:** 10.1186/s10194-022-01470-4

**Published:** 2022-08-18

**Authors:** Christian Lampl, Viktoria Kraus, Katrina Lehner, Brett Loop, Mahan Chehrenama, Zofia Maczynska, Shannon Ritter, Jan Klatt, Josefin Snellman

**Affiliations:** 1 Department of Neurology, Konventhospital Barmherzige Brüder Linz, Seilerstätte 2, 4020 Linz, Austria; 2 Novartis Pharmaceuticals, Cambridge, MA USA; 3 Amgen Inc, Thousand Oaks, CA USA; 4 Novartis Pharma AG, Basel, Switzerland; 5 Novartis Pharmaceutical Corporation, East Hanover, NJ USA; 6 Merck KgaA, Darmstadt, Germany

**Keywords:** Advanced age, Calcitonin gene-related peptide, Cardiovascular, Cerebrovascular, Older individuals, Erenumab, Gastrointestinal, Monoclonal antibody, Randomized controlled trial, Safety, Tolerability

## Abstract

**Background:**

Erenumab, a fully human monoclonal antibody that targets the calcitonin gene-related peptide receptor, has demonstrated efficacy and safety in the prevention of episodic and chronic migraine. There exists an unmet need to establish the safety of erenumab in older individuals, in view of existing multiple comorbidities, polypharmacy, and age-related physiological changes. This pooled analysis of five large migraine-prevention studies examined the safety of erenumab stratified across age groups, particularly in older populations.

**Methods:**

Pooled and age-stratified analysis of safety data from the 12-week double-blind treatment phase (DBTP) of five randomized, placebo-controlled Phase 2 and 3 studies of erenumab in participants with episodic or chronic migraine across the age groups < 40 years, 40–49 years, 50–59 years, and ≥ 60 years was completed. The safety of erenumab across age groups was determined by assessing safety endpoints including treatment-emergent adverse events (AEs), serious AEs, and events leading to study drug discontinuation.

**Results:**

Overall, 3345 participants across five studies were randomized to receive either placebo (*n* = 1359), erenumab 70 mg (*n* = 1132) or erenumab 140 mg (*n* = 854); 3176 (94.9%) completed the DBTP, and 169 (5.1%) discontinued, mainly due to participant decision (110; 3.3%). Overall, 1349 (40.6%), 1122 (33.8%), and 850 (25.6%) participants received at least one dose of placebo, erenumab 70 mg, and erenumab 140 mg, respectively.

Incidence of treatment-emergent AEs was similar across all age groups for both doses of erenumab (70 mg or 140 mg) and placebo (< 40 years, 44.0% vs 44.4%; 40–49 years, 42.5% vs 49.2%; 50–59 years, 46.5% vs 41.6%; ≥ 60 years, 43.8% vs 59.4%). Incidence of treatment-emergent serious AEs overall, and stratified by age groups for both doses and placebo was low (< 40 years, 0.9% vs 1.2%; 40–49 years, 1.7% vs 1.9%; and 50–59 years, 1.6% vs 1.1%), with no serious AEs reported in participants aged ≥ 60 years. No deaths were reported.

**Conclusions:**

Erenumab (70 mg or 140 mg) exhibited a similar safety profile compared with placebo across age groups in individuals with episodic or chronic migraine, with no increased emergence of events due to age. Erenumab was well tolerated in older participants with multiple comorbidities, polypharmacy, and age-related physiological changes.

**Trial registration number:**

ClinicalTrials.gov Identifiers: NCT02066415, NCT02456740, NCT02483585, NCT03096834, NCT03333109.

**Supplementary Information:**

The online version contains supplementary material available at 10.1186/s10194-022-01470-4.

## Introduction

Migraine is a disabling neurological disease affecting all ages [1] and is the second leading cause of years lived with disability worldwide [2]. Studies have shown that migraine has a substantial impact on individuals in terms of quality of life (QoL) and imposes a heavy socioeconomic burden, with the majority of direct costs due to a higher utilization of healthcare resources compared with matched individuals without migraine.

The prevalence of migraine peaks in those aged 30‒39 years and is relatively lower at the end of the lifespan [3, 4], with a one-year prevalence of approximately 10% in older age groups [5]. As life expectancy increases worldwide, it is likely that a larger percentage of patients will be living with migraine as they grow older [5, 6]. Treatment can become more challenging in these individuals due to the need to consider potential age-related changes in medication metabolism and increased medical comorbidities. For example, older individuals are more likely to experience polypharmacy due to comorbid medical conditions such as hypertension, heart disease, and stroke [5]. Age can also lead to physiological changes that may directly impact the pharmacokinetics and pharmacodynamics of drugs [5]. Such age-related factors may affect the effectiveness of migraine medications as well as their tolerability profile.

The preventive treatment of migraine has typically involved the use of oral medications, such as beta-blockers (propranolol), anti-epileptics (topiramate), and tricyclic anti-depressants (amitriptyline), that were originally developed for other conditions and subsequently repurposed for migraine. Medication side effects associated with these treatments, leading to poor patient adherence, [7–10] may therefore be a another limiting factor when considering treatment options. Thus, the need for migraine specific prophylactic treatment, specifically targeting older individuals, is of growing importance in order to reduce the risk of medication overuse and most importantly, to increase their QoL.

Recent advances in the field of migraine have led to the development of disease-specific preventive medications. Monoclonal antibodies targeting the calcitonin gene-related peptide (CGRP) receptor (erenumab) or CGRP directly (eptinezumab, fremanezumab, and galcanezumab) are new therapeutic agents for the preventive treatment of migraine. They represent an extension of the therapeutic options, which already exist for migraine prevention. Erenumab, a fully human monoclonal antibody, is an approved potent and selective CGRP receptor antagonist specifically designed to prevent migraine [11]. The clinical efficacy and safety of erenumab has been established in several placebo-controlled studies in episodic migraine (EM) and chronic migraine (CM), [12–17] including a 5-year open-label study in EM [18]. While previous pooled studies of large clinical trials have analyzed the cardiovascular (CV) [19] and non-CV [20] risk associated with erenumab, an age-stratified analysis has never been performed due to a lack of data for the older age group.

Thus, the aim of this pooled analysis was to examine the safety and tolerability of erenumab in adults with EM or CM across different age groups (< 40, 40–49, 50–59, and ≥ 60 years) using pooled data from five large randomized, placebo-controlled trials. This pooled analysis evaluated CV, cerebrovascular and gastrointestinal (GI) adverse events (AEs) as AEs of special interest (AESI) to help physicians make an informed treatment choice of migraine therapy in individuals of older age.

## Methods

### Study design

**Fig. 1:**
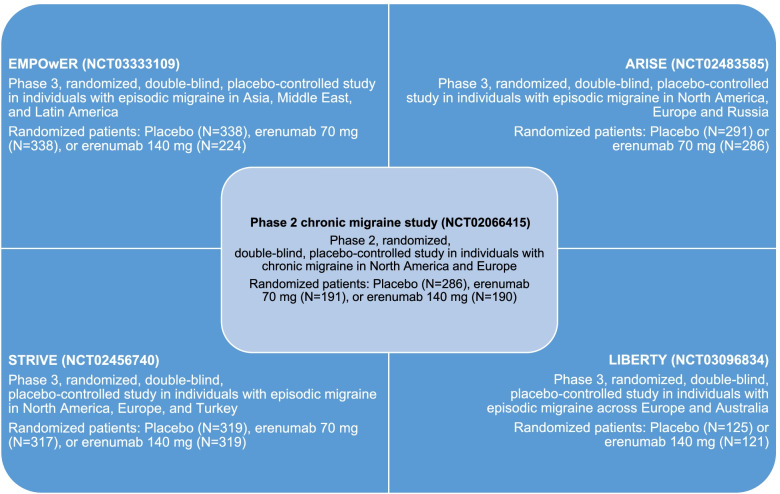
Summary of phase 2 and 3 studies included in the pooled analysis

This pooled analysis evaluated the safety and tolerability of erenumab versus placebo in individuals with migraine from five randomized, double-blind, placebo-controlled studies (Fig. 1). The pool comprised a phase 2 CM study (ClinicalTrials.gov number NCT02066415) [15] and four phase 3 EM studies (ClinicalTrials.gov numbers NCT02456740, STRIVE; NCT02483585, ARISE; NCT03096834, LIBERTY; and NCT03333109, EMPOwER) [13, 14, 16, 17].

All studies included a screening phase of 3 weeks (except for LIBERTY and EMPOwER, which had a shorter screening phase of 2 weeks), a baseline phase of 4 weeks, a double-blind treatment phase (DBTP) of 12 weeks (except for STRIVE study, which had a DBTP of 24 weeks), followed by a 12-week safety follow-up phase. The STRIVE study also included an active-treatment phase during which participants underwent repeat randomization and were assigned to receive erenumab 70 mg or 140 mg for an additional 28 weeks. Safety data from the 12-week DBTP of each study were included in this pooled analysis. In all five studies, the investigational drug product (erenumab 70 mg, erenumab 140 mg, or placebo) was subcutaneously administered once monthly.

The integrated data utilized the safety analysis set of each study, which included all randomized participants who had received at least one dose of study drug (erenumab 70 mg, erenumab 140 mg, or placebo). Data stratification was done according to the following age groups: < 40, 40–49, 50–59, and ≥ 60 years.

### Participants

The detailed inclusion and exclusion criteria of the individual studies have been published previously. Briefly, eligible participants were adults aged 18–65 years with a history of CM, with or without aura (phase 2 study); or EM, with or without aura for ≥ 12 months (phase 3 studies). Although the inclusion criterion in all studies comprised individuals aged between 18 to 65 years, there was one participant aged 17 years and one aged 66 years in the data pool.

Participants were permitted to use acute headache treatments including migraine-specific medications (i.e., triptans, ergotamine derivatives) and nonsteroidal anti- inflammatory drugs during the phase 2 CM study, and STRIVE, LIBERTY, and ARISE studies.

### Safety endpoints and analysis

Safety endpoints included the incidence of overall AEs, serious AEs, treatment-related AEs, and AEs leading to study drug discontinuation. Only laboratory abnormalities reported as an AE (considered clinically significant by investigators) were analyzed.

Treatment-emergent adverse events (TEAEs) with an onset day within the first 3 months (91 days) from the first administration of erenumab (70 mg or 140 mg) or placebo were summarized. Standardized search terms were used to identify AESIs from the CV, cerebrovascular, and GI system organ class (SOC).

An AE was defined as any unfavorable and unintended sign, symptom or disease temporarily associated with the use of study drug that may or may not be related. A serious AE was defined as an AE that met at least one of the following criteria: fatal, life-threatening, required inpatient hospitalization or prolongation of existing hospitalization, resulted in persistent or significant disability/incapacity, congenital anomaly/birth defect, or another medically important serious event. TEAE was defined as an event that emerged during treatment, having been absent pre-treatment or had worsened relative to the pre-treatment state [21]. An adverse drug reaction or treatment-related AE was defined as a noxious and unintended response to study drug, which occurred at doses normally used in human subjects for prophylaxis, diagnosis, or therapy of disease or for modification of physiological function. AESIs (serious or non-serious) were defined as AEs of scientific/medical concern specific to study drug, for which ongoing monitoring and rapid communication to other parties (e.g. regulators) might be warranted. AEs leading to study drug discontinuation were defined as any AE (serious or non-serious) that led to the permanent withdrawal of study treatment prior to the defined study treatment completion date.

The Medical Dictionary for Regulatory Activities (MedDRA) was used to code AEs according to the preferred term (PT) and SOC. MedDRA version 19.0 was used for the phase 2 CM, ARISE, and STRIVE studies, v20.1 for the LIBERTY study, and v22.0 for the EMPOwER study. AE severity was graded using the Common Terminology Criteria for Adverse Events (CTCAE) criteria (v4.0 for phase 2 CM and STRIVE, v4.03 for ARISE, LIBERTY, and EMPOwER), whereby Grade 1 AEs were considered mild, Grade 2 AEs were considered moderate, Grade 3 AEs were considered severe or medically significant but not immediately life-threatening, and Grade 4 AEs were considered life-threatening.

Laboratory values or test results were considered as AEs only if they fulfilled at least one of the following: they induced clinical signs or symptoms, they were considered clinically significant, or they required therapy. Clinically significant laboratory values or test results for individual patients were evaluated by study investigators to determine if they fell outside defined normal ranges, represented significant changes from baseline or a previous visit, or were considered non-typical in a patient with underlying disease. Laboratory tests conducted included tests of hepatic function, hematology, clinical chemistry, and urinalysis. Laboratory results identified as AEs were graded according to the same criteria as all other AEs.

### Ethical considerations

All studies in this pooled analysis were conducted in compliance with the Declaration of Helsinki, International Council for Harmonization Guidelines for Good Clinical Practice, and local country regulations. The study protocols were approved by the Institutional Review Board (IRB) or Independent Ethics Committee (IEC) at each center. A list of the IEC or IRB for each study has been provided in the Additional file 1. Individuals provided written informed consent prior to initiation of any study procedures. Site investigators collected the data and Novartis conducted the data analyses.

### Statistical analyses

The safety analysis set included all participants who received at least one dose of erenumab (70 mg or 140 mg) or placebo during the DBTP. The AE summary contained TEAEs with onset day within the first 3 months (91 days) from the first administration of either erenumab or placebo. Exposure-adjusted incidence rates (per 100 patient-years) of AEs were calculated by dividing the total number of participants who reported at least one AE by total time at risk (in years, summed across all participants), and multiplying by 100. Total time at risk in the DBTP was the time from first administration of the study drug until the 91^st^ day after the first drug administration or onset of first event. The overall number of patients included in both erenumab doses (70 mg and 140 mg) may not sum up to the number in the total number of patients receiving any dose of erenumab column, as there were patients who received both doses throughout the study. However, the AEs displayed in the tables in this manuscript always refer to the dose at which the event occurred.

## Results

### Participants

Overall, 3345 participants were randomized across the five double-blind placebo-controlled studies and were included in the pooled analysis. Demographic and baseline characteristics of the 3345 participants are summarized in Table 1 (stratified by age groups) and in Additional file 1 (overall population).

**Table 1:** Demographics and baseline characteristics stratified by age group

**Aged < 40 years**	**Placebo (*n*** **= 600)**	**Erenumab 70 mg (*n*** **= 517)**	**Erenumab 140 mg (*n*** **= 383)**	**Total (*N*** **= 1500)**
**Age**, mean (SD)^a^	30.8 (5.8)	30.2 (6.1)	30.6 (5.7)	30.5 (5.9)
**Female**, n (%)	495 (82.5)	439 (84.9)	316 (82.5)	1250 (83.3)
**Race**, n (%)				
Asian	161 (26.8)	172 (33.3)	116 (30.3)	449 (29.9)
White	384 (64.0)	295 (57.1)	233 (60.8)	912 (60.8)
**BMI** (kg/m^2^), mean (SD)	26.1 (6.0)	26.1 (6.0)	25.9 (6.0)	26.0 (7.0)
**MMDs**, mean (SD)	10.4 (5.2)	9.7 (4.7)	10.2 (5.4)	10.1 (5.1)
**MMDs, categories**, n (%)				
8–14 MMDs	279 (46.5)	238 (46.0)	181 (47.3)	698 (46.5)
≥ 15 MMDs	96 (16.0)	67 (13.0)	46 (12.0)	209 (13.9)
**Medication overuse**, n (%)^b^				
Yes	37 (6.2)	29 (5.6)	23 (6.0)	89 (5.9)
No	77 (12.8)	48 (9.3)	42 (11.0)	167 (11.1)
**PPTF**, n (%)				
**0**	363 (60.5)	337 (65.2)	211 (55.1)	911 (60.7)
1	102 (17.0)	96 (18.6)	70 (18.3)	268 (17.9)
2	48 (8.0)	41 (7.9)	46 (12.0)	135 (9.0)
≥ 3	87 (14.5)	43 (8.3)	56 (14.6)	186 (12.4)
**Framingham cardiovascular risk factors**				
**Cigarette use**, n (%)				
Current	30 (5.0)	36 (7.0)	26 (6.8)	92 (6.1)
Former	33 (5.5)	28 (5.4)	10 (2.6)	71 (4.7)
Never	195 (32.5)	177 (34.2)	108 (28.2)	480 (32.0)
Unknown	342 (57.0)	276 (53.4)	239 (62.4)	857 (57.1)
**Diabetes**, n (%)	3 (0.5)	4 (0.8)	3 (0.8)	10 (0.7)
**Hypertension**, n (%)	39 (6.5)	22 (4.3)	18 (4.7)	79 (5.3)
**Total cholesterol** (mmol/L), mean (SD)	4.6 (1.0)	4.6 (0.8)	4.6 (1.0)	4.6 (0.8)
**HDL cholesterol** (mmol/L), mean (SD)	1.4 (0.4)	1.4 (0.4)	1.4 (0.4)	1.4 (0.4)
**Systolic BP** (mmHg), mean (SD)	116.9 (11.9)	115.4 (11.4)	115.7 (11.6)	116.1 (12.0)
**Coronary artery disease**, n (%)	0 (0.0)	0 (0.0)	0 (0.0)	0 (0.0)
**Cerebrovascular or peripheral artery disease**, n (%)	1 (0.2)	0 (0.0)	2 (0.5)	3 (0.2)
**Aged 40–49 years**	**Placebo (*n* = 426)**	**Erenumab 70 mg (*n* = 361)**	**Erenumab 140 mg (*n* = 278)**	**Total (*N* = 1065)**
**Age**, mean (SD)	44.4 (2.8)	44.4 (2.8)	44.6 (2.8)	44.4 (2.8)
**Female**, n (%)	361 (84.7)	303 (83.9)	237 (85.3)	901 (84.6)
**Race**, n (%)				
Asian	78 (18.3)	82 (22.7)	45 (16.2)	205 (19.2)
White	317 (74.4)	259 (71.7)	224 (80.6)	800 (75.1)
**BMI** (kg/m^2^), mean (SD)	26.6 (5.3)	26.3 (5.3)	26.1 (4.9)	26.4 (5.2)
**MMDs**, mean (SD)	10.3 (4.7)	9.7 (4.4)	10.9 (4.8)	10.2 (4.6)
**MMDs, categories**, n (%)				
8–14 MMDs	224 (52.6)	186 (51.5)	141 (50.7)	551 (51.7)
≥ 15 MMDs	63 (14.8)	40 (11.1)	52 (18.7)	155 (14.6)
**Medication overuse**, n (%)^b^				
Yes	42 (9.9)	31 (8.6)	27 (9.7)	100 (9.4)
No	49 (11.5)	33 (9.1)	43 (15.5)	125 (11.7)
**PPTF**, n (%)				
0	182 (42.7)	200 (55.4)	122 (43.9)	504 (47.3)
1	90 (21.1)	67 (18.6)	36 (12.9)	193 (18.1)
2	68 (16.0)	47 (13.0)	61 (21.9)	176 (16.5)
≥ 3	86 (20.2)	47 (13.0)	59 (21.2)	192 (18.0)
**Framingham cardiovascular risk factors**				
**Cigarette use**, n (%)	17 (4.0)	20 (5.5)	9 (3.2)	46 (4.3)
Current	39 (9.2)	32 (8.9)	18 (6.5)	89 (8.4)
Former	135 (31.7)	145 (40.2)	75 (27.0)	355 (33.3)
Never	235 (55.2)	164 (45.4)	176 (63.3)	575 (54.0)
Unknown	9 (2.1)	8 (2.2)	3 (1.1)	20 (1.9)
**Diabetes**, n (%)	55 (12.9)	41 (11.4)	35 (12.6)	131 (12.3)
**Hypertension**, n (%)	17 (4.0)	20 (5.5)	9 (3.2)	46 (4.3)
**Total cholesterol** (mmol/L), mean (SD)	5.1 (0.9)	5.0 (0.9)	5.0 (0.9)	5.0 (0.9)
**HDL cholesterol** (mmol/L), mean (SD)	1.5 (0.4)	1.5 (0.4)	1.5 (0.4)	1.5 (0.4)
**Systolic BP** (mmHg), mean (SD)	120.0 (12.4)	119.0 (13.0)	119.6 (13.2)	119.5 (12.8)
**Coronary artery disease**, n (%)	2 (0.5)	1 (0.3)	0 (0.0)	3 (0.3)
**Cerebrovascular or peripheral artery disease**, n (%)	4 (0.9)	1 (0.3)	1 (0.4)	6 (0.6)
**Aged 50–59 years**	**Placebo (*n* = 263)**	**Erenumab 70 mg (*n* = 216)**	**Erenumab 140 mg (*n* = 158)**	**Total (*N* = 637)**
**Age**, mean (SD)	53.9 (2.8)	53.9 (2.8)	53.7 (2.8)	53.8 (2.8)
**Female**, n (%)	220 (83.7)	178 (82.4)	133 (84.2)	531 (83.4)
**Race**, n (%)				
Asian	36 (13.7)	23 (10.6)	15 (9.5)	74 (11.6)
White	208 (79.1)	179 (82.9)	128 (81.0)	515 (80.8)
**BMI** (kg/m^2^), mean (SD)	25.8 (5.3)	26.8 (5.3)	25.7 (4.9)	26.1 (5.2)
**MMDs**, mean (SD)	10.9 (5.6)	10.2 (4.9)	10.8 (5.1)	10.6 (5.3)
**MMDs, categories**, n (%)				
8–14 MMDs	117 (44.5)	98 (45.4)	67 (42.4)	282 (44.3)
≥ 15 MMDs	51 (19.4)	37 (17.1)	39 (24.7)	127 (19.9)
**Medication overuse**, n (%)^b^				
Yes	31 (11.8)	15 (6.9)	25 (15.8)	71 (11.1)
No	30 (11.4)	28 (13.0)	22 (13.9)	80 (12.6)
**PPTF**, n (%)				
0	115 (43.7)	101 (46.8)	67 (42.4)	283 (44.4)
1	55 (20.9)	54 (25.0)	21 (13.3)	130 (20.4)
2	40 (15.2)	25 (11.6)	21 (13.3)	86 (13.5)
≥ 3	53 (20.2)	36 (16.7)	49 (31.0)	138 (21.7)
**Framingham cardiovascular risk factors**				
**Cigarette use**, n (%)				
Current	14 (5.3)	11 (5.1)	6 (3.8)	31 (4.9)
Former	28 (10.6)	31 (14.4)	20 (12.7)	79 (12.4)
Never	81 (30.8)	93 (43.1)	35 (22.2)	209 (32.8)
Unknown	140 (53.2)	81 (37.5)	97 (61.4)	318 (49.9)
**Diabetes**, n (%)	13 (4.9)	7 (3.2)	3 (1.9)	23 (3.6)
**Hypertension**, n (%)	56 (21.3)	40 (18.5)	25 (15.8)	121 (19.0)
**Total cholesterol** (mmol/L), mean (SD)	5.5 (1.0)	5.3 (1.0)	5.4 (1.0)	5.4 (1.0)
**HDL cholesterol** (mmol/L), mean (SD)	1.6 (0.5)	1.6 (0.5)	1.6 (0.5)	1.6 (0.5)
**Systolic BP** (mmHg), mean (SD)	121.1 (14.8)	122.2 (14.2)	122.1 (13.8)	121.7 (14.4)
**Coronary artery disease**, n (%)	0 (0.0)	0 (0.0)	2 (1.3)	2 (0.3)
**Cerebrovascular or peripheral artery disease**, n (%)	0 (0.0)	2 (0.9)	1 (0.6)	3 (0.5)
**Aged** ≥ **60 years**	**Placebo (*n* = 70)**	**Erenumab 70 mg (*n* = 38)**	**Erenumab 140 mg (*n* = 35)**	**Total (*N* = 143)**
**Age**, mean (SD)^a^	62.0 (1.6)	62.1 (1.5)	61.7 (1.6)	61.9 (1.6)
**Female**, n (%)	55 (78.6)	31 (81.6)	27 (77.1)	113 (79.0)
**Race**, n (%)				
Asian	2 (2.9)	3 (7.9)	4 (11.4)	9 (6.3)
White	65 (92.9)	33 (86.8)	31 (88.6)	129 (90.2)
**BMI** (kg/m^2^), mean (SD)	26.3 (4.8)	25.4 (5.6)	26.1 (4.9)	26.0 (5.0)
**MMDs**, mean (SD)	11.2 (5.5)	10.7 (5.3)	10.8 (3.7)	11.0 (5.0)
**MMDs, categories**, n (%)				
8–14 MMDs	36 (51.4)	22 (57.9)	22 (62.9)	80 (55.9)
≥ 15 MMDs	14 (20.0)	5 (13.2)	5 (14.3)	24 (16.8)
**Medication overuse**, n (%)^b^				
Yes	7 (10.0)	4 (10.5)	3 (8.6)	14 (9.8)
No	13 (18.6)	3 (7.9)	5 (14.3)	21 (14.7)
**PPTF**, n (%)				
0	26 (37.1)	17 (44.7)	14 (40.0)	57 (39.9)
1	11 (15.7)	7 (18.4)	4 (11.4)	22 (15.4)
2	12 (17.1)	7 (18.4)	5 (14.3)	24 (16.8)
≥ 3	21 (30.0)	7 (18.4)	12 (34.3)	40 (28.0)
**Framingham cardiovascular risk factors**				
**Cigarette use**, n (%)				
Current	3 (4.3)	0 (0.0)	0 (0.0)	3 (2.1)
Former	15 (21.4)	4 (10.5)	3 (8.6)	22 (15.4)
Never	20 (28.6)	24 (63.2)	9 (25.7)	53 (37.1)
Unknown	32 (45.7)	10 (26.3)	23 (65.7)	65 (45.5)
**Diabetes**, n (%)	1 (1.4)	1 (2.6)	1 (2.9)	3 (2.1)
**Hypertension**, n (%)	19 (27.1)	6 (15.8)	9 (25.7)	34 (23.8)
**Total cholesterol** (mmol/L), mean (SD)	5.3 (1.1)	5.35 (0.8)	5.6 (1.0)	5.4 (1.0)
**HDL cholesterol** (mmol/L), mean (SD)	1.6 (0.4)	1.8 (0.5)	1.5 (0.4)	1.6 (0.5)
**Systolic BP** (mmHg), mean (SD)	124.1 (12.9)	120.6 (14.3)	124.2 (15.4)	123.2 (13.9)
**Coronary artery disease**, n (%)	1 (1.4)	0 (0.0)	0 (0.0)	1 (0.7)
**Cerebrovascular or peripheral artery disease**, n (%)	3 (4.3)	0 (0.0)	2 (5.7)	5 (3.5)

Smoking-related information was not collected in the LIBERTY and EMPOwER studies. Hence, these were counted under the “Unknown” category in the parameter “Cigarette use”. Total cholesterol and HDL-related information was not collected in the LIBERTY study. Data were pooled from the Phase 2 CM (randomized analysis set), STRIVE (full analysis set), ARISE (full analysis set), LIBERTY (randomized analysis set), and EMPOwER (randomized analysis set) studies and are presented as mean (SD), unless stated

Abbreviations: *BMI* Body mass index, *BP* Blood pressure, *CM* Chronic migraine, *EM* Episodic migraine, *HDL* High-density lipoprotein, *MMD* Monthly migraine days, *PPTF* Prior preventive treatment failure, *SD* Standard deviation

^a^Although the inclusion criterion in all studies was age between 18 to 65 years, there was one patient aged 17 years and one aged 66 years in the data pool

^b^Medication overuse was captured only for phase 2 CM study

The mean age of overall participants was 40.7 years; 83.6% were females (*n* = 2795), and 70.4% were white (*n* = 2356). The overall population reported 10.3 (SD ± 4.98) mean monthly migraine days (MMDs), 8.2% reported medication overuse, and 16.6% reported ≥ 3 prior preventive treatment failures (for age stratification please see Table 1). Nearly half (*n* = 1611, 48.2%) of the overall population reported 8–14 MMDs at baseline, while only 515 (15.4%) participants reported ≥ 15 MMDs. In total, 1755 (52.5%) participants reported no prior treatment failure. Older participants (≥ 60 years) reported a higher number of prior preventive treatment failures (PPTFs) compared with younger participants (< 40 years); the number of participants who reported ≥ 3 PPTFs was higher in the older age group (overall population, 556 [16.6%]; participants aged < 40 years, 186 [12.4%]; 40–49 years, 192 [18.0%]; 50–59 years, 138 [21.7%]; and ≥ 60 years, 40 [28.0%]).

Framingham CV risk factors are presented in Table 1 (stratified by age groups) and in Additional file 1 (overall population). At study baseline, nearly one-third (32.8%) of participants were non-smokers, 7.8% were former smokers, and 5.1% were current smokers (Additional file 1). In the overall population, incidence of diabetes (1.7%), coronary artery disease (0.2%), and cerebrovascular or peripheral artery disease (0.5%) was low, while 10.9% of participants had a prior history of hypertension. Although the preexisting medical history for vascular risk factors was low, there was a trend of increasing incidence of diabetes (2.1%), hypertension (23.8%), coronary artery disease (0.7%), and cerebrovascular or peripheral artery disease (3.5%) in older participants (≥ 60 years), which is typical for this age group (Additional file 1) [5].

As expected, the number of participants with comorbid conditions increased with age, starting with 75.7% aged < 40 years, 87.0% aged between 40–49 years, 92.3% aged 50–59 years, and 94.4% aged ≥ 60 years. Commonly reported comorbid conditions included migraine with aura (45.8%), migraine without aura (45.8%), anxiety (46.6%), depression (47.3%), and seasonal allergy (12.4%). Comorbid conditions were generally well balanced across treatment groups and age groups. Interestingly, comorbidities seemed to be higher in the erenumab 70 mg group across all age groups (data not shown).

### Disposition

**Fig. 2:**
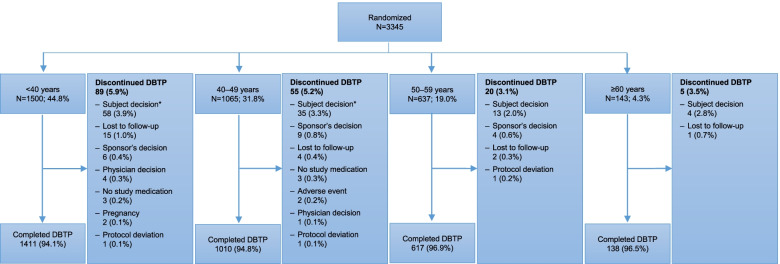
Disposition of participants. *Subject decision was a sum of subject decision and subject/guardian decision. In the EMPOwER study, six randomized participants did not take the study medication and were not formally entered into the DBTP; these participants are counted under the “no study medication” category. Abbreviation: DBTP, double-blind treatment phase

Overall, across the pooled analysis, 3176/3345 (94.9%) participants completed the DBTP and 169/3345 (5.1%) discontinued. Age did not influence the completion status of the DBTP (Fig. 2). The primary reason for discontinuation was participant decision (*n* = 110, 3.3%).

### Exposure

In total, 1972 participants received at least one dose of erenumab (1122 received 70 mg and 850 received 140 mg). Most participants received three doses of erenumab over the DBTP (*n* = 1895; of which 1075 received erenumab 70 mg, and 820 received 140 mg); 45 participants received two doses of erenumab, and 32 received one dose. Overall, the mean duration of exposure to either dose of erenumab (70 mg or 140 mg) was 11.9 weeks, and a total exposure of 449.2 patient-years to erenumab (70 mg or 140 mg).

### Safety analysis

#### Overall TEAEs

A summary of TEAEs by age group is presented in Table 2. Overall, 622/1349 (46.1%) participants who received placebo and 867/1972 (44.0%) participants who received erenumab reported at least one TEAE during the DBTP. The incidence of TEAEs by age group was similar in either dose group (70 mg or 140 mg): < 40 years, 44.0%; 40–49 years, 42.5%; 50–59 years, 46.5%; ≥ 60 years, 43.8%.

**Table 2:** Summary of TEAEs by age groups (safety analysis set, double-blind treatment phase)

**Aged < 40 years, n (%)**	**Placebo (*n*** **= 597)**	**Erenumab 70 mg (*n*** **= 514)**	**Erenumab 140 mg (*n*** **= 382)**	**Erenumab 70 + 140 mg (*n*** **= 896)**	**Total (*N*** **= 1493)**
All TEAEs	265 (44.4)	227 (44.2)	167 (43.7)	394 (44.0)	659 (44.1)
Grade ≥ 2	135 (22.6)	106 (20.6)	65 (17.0)	171 (19.1)	306 (20.5)
Grade ≥ 3	12 (2.0)	13 (2.5)	6 (1.6)	19 (2.1)	31 (2.1)
Grade ≥ 4	0	0	0	0	0
SAEs	7 (1.2)	6 (1.2)	2 (0.5)	8 (0.9)	15 (1.0)
AEs leading to discontinuation of study drug	5 (0.8)	6 (1.2)	4 (1.0)	10 (1.1)	15 (1.0)
Fatal AEs	0	0	0	0	0
Treatment-related AEs	66 (11.1)	71 (13.8)	48 (12.6)	119 (13.3)	185 (12.4)
**Aged 40–49 years, n (%)**	**Placebo (*n*** **= 421)**	**Erenumab 70 mg (*n*** **= 356)**	**Erenumab 140 mg (*n*** **= 275)**	**Erenumab 70 + 140 mg (*n*** **= 631)**	**Total (*N*** **= 1052)**
All TEAEs	207 (49.2)	150 (42.1)	118 (42.9)	268 (42.5)	475 (45.2)
Grade ≥ 2	104 (24.7)	72 (20.2)	51 (18.5)	123 (19.5)	227 (21.6)
Grade ≥ 3	11 (2.6)	11 (3.1)	9 (3.3)	20 (3.2)	31 (2.9)
Grade ≥ 4	0	1 (0.3)	0	1 (0.2)	1 (0.1)
SAEs	8 (1.9)	6 (1.7)	5 (1.8)	11 (1.7)	19 (1.8)
AEs leading to discontinuation of study drug	3 (0.7)	5 (1.4)	3 (1.1)	8 (1.3)	11 (1.0)
Fatal AEs	0	0	0	0	0
Treatment-related AEs	54 (12.8)	47 (13.2)	27 (9.8)	74 (11.7)	128 (12.2)
**Aged 50–59 years, n (%)**	**Placebo (*n*** **= 262)**	**Erenumab 70 mg (*n*** **= 214)**	**Erenumab 140 mg (*n*** **= 158)**	**Erenumab 70 + 140 mg (*n*** **= 372)**	**Total (*N*** **= 634)**
All TEAEs	109 (41.6)	93 (43.5)	80 (50.6)	173 (46.5)	282 (44.5)
Grade ≥ 2	54 (20.6)	43 (20.1)	43 (27.2)	86 (23.1)	140 (22.1)
Grade ≥ 3	6 (2.3)	6 (2.8)	5 (3.2)	11 (3.0)	17 (2.7)
Grade ≥ 4	0	0	0	0	0
SAEs	3 (1.1)	4 (1.9)	2 (1.3)	6 (1.6)	9 (1.4)
AEs leading to discontinuation of study drug	2 (0.8)	2 (0.9)	3 (1.9)	5 (1.3)	7 (1.1)
Fatal AEs	0	0	0	0	0
Treatment-related AEs	19 (7.3)	28 (13.1)	20 (12.7)	48 (12.9)	67 (10.6)
**Aged ≥ 60 years, n (%)**	**Placebo (*n*** **= 69)**	**Erenumab 70 mg (*n*** **= 38)**	**Erenumab 140 mg (*n*** **= 35)**	**Erenumab 70 + 140 mg (*n*** **= 73)**	**Total (*N*** **= 142)**
All TEAEs	41 (59.4)	15 (39.5)	17 (48.6)	32 (43.8)	73 (51.4)
Grade ≥ 2	23 (33.3)	11 (28.9)	9 (25.7)	20 (27.4)	43 (30.3)
Grade ≥ 3	4 (5.8)	0	1 (2.9)	1 (1.4)	5 (3.5)
Grade ≥ 4	0	0	1 (2.9)	1 (1.4)	1 (0.7)
SAEs	0	0	0	0	0
AEs leading to discontinuation of study drug	1 (1.4)	0	1 (2.9)	1 (1.4)	2 (1.4)
Fatal AEs	0	0	0	0	0
Treatment-related AEs	9 (13.0)	6 (15.8)	3 (8.6)	9 (12.3)	18 (12.7)

Data pooled from the phase 2 CM, STRIVE, ARISE, LIBERTY, and EMPOwER studies (safety analysis set). The summary contains TEAEs with onset day within the first 3 months (91 days) from the first administration of erenumab/placebo

Abbreviations: *AE* Adverse event, *CM* Chronic migraine, *SAE* Serious adverse event, *TEAE* Treatment-emergent adverse event

A slightly higher incidence rate of TEAEs was observed in participants in the 50- to 59-year age group receiving 140 mg erenumab (80/158; 50.6%) compared with other age groups. A similar higher incidence rate of TEAEs was observed in participants in the ≥ 60-year age group receiving placebo (41/69; 59.4%). These differences are presumed to be normal variations and not driven by CV, cerebrovascular, and/or GI AEs (that are most common in older patients) or linked to any specific class of AEs (Table 2).

Most AEs were Grade 1 or Grade 2. Incidence of Grade 2 TEAEs was similar for both erenumab doses (70 mg or 140 mg) stratified by age group: < 40 years, 19.1%; 40–49 years, 19.5%; 50–59 years, 23.1%; ≥ 60 years, 27.4%. One participant receiving erenumab 70 mg (in the 40- to 49-year age group) and one participant receiving 140 mg (in the ≥ 60-year age group) reported one Grade 4 TEAE each.

No deaths were reported in any study during the DBTP. Incidence of treatment-related TEAEs was also balanced across both erenumab doses (70 mg or 140 mg) stratified by age group: < 40 years, 13.3%; 40–49 years, 11.7%; 50–59 years, 12.9%; ≥ 60 years, 12.3% (Table 2).

The most commonly reported TEAEs, occurring in ≥ 3.0% of participants in any age group, were viral upper respiratory tract infections and upper respiratory tract infections, which were common in all treatment groups (Table 3). In addition, participants aged ≥ 60 years reported fatigue, alopecia, back pain, ligament sprain, musculoskeletal stiffness, and sinusitis.

**Table 3:** TEAEs with incidence of ≥ 3.0% in any erenumab group (safety analysis set), by preferred term

**Aged < 40 years, n (%)**	**Placebo (*n*** **= 597)**	**Erenumab 70 mg (*n*** **= 514)**	**Erenumab 140 mg (*n*** **= 382)**	**Erenumab 70 + 140 mg (*n*** **= 896)**	**Total (*N*** **= 1493)**
**Number of participants with at least one AE**	265 (44.4)	227 (44.2)	167 (43.7)	394 (44.0)	659 (44.1)
Upper respiratory tract infection	19 (3.2)	23 (4.5)	15 (3.9)	38 (4.2)	57 (3.8)
Viral upper respiratory tract infection	24 (4.0)	23 (4.5)	14 (3.7)	37 (4.1)	61 (4.1)
Constipation	6 (1.0)	16 (3.1)	15 (3.9)	31 (3.5)	37 (2.5)
Injection-site pain	10 (1.7)	22 (4.3)	9 (2.4)	31 (3.5)	41 (2.7)
**Aged 40–49 years, n (%)**	**Placebo (*n*** **= 421)**	**Erenumab 70 mg (*n*** **= 356)**	**Erenumab 140 mg (*n*** **= 275)**	**Erenumab 70 + 140 mg (*n*** **= 631)**	**Total (*N*** **= 1052)**
**Number of participants with at least one AE**	207 (49.2)	150 (42.1)	118 (42.9)	268 (42.5)	475 (45.2)
Viral upper respiratory tract infection	23 (5.5)	15 (4.2)	8 (2.9)	23 (3.6)	46 (4.4)
Upper respiratory tract infection	8 (1.9)	12 (3.4)	2 (0.7)	14 (2.2)	22 (2.1)
**Aged 50–59 years, n (%)**	**Placebo (*n*** **= 262)**	**Erenumab 70 mg (*n*** **= 214)**	**Erenumab 140 mg (*n*** **= 158)**	**Erenumab 70 + 140 mg (*n*** **= 372)**	**Total (*N*** **= 634)**
**Number of participants with at least one AE**	109 (41.6)	93 (43.5)	80 (50.6)	173 (46.5)	282 (44.5)
Viral upper respiratory tract infection	11 (4.2)	10 (4.7)	10 (6.3)	20 (5.4)	31 (4.9)
Upper respiratory tract infection	6 (2.3)	9 (4.2)	4 (2.5)	13 (3.5)	19 (3.0)
Constipation	3 (1.1)	4 (1.9)	7 (4.4)	11 (3.0)	14 (2.2)
Injection-site pain	2 (0.8)	3 (1.4)	5 (3.2)	8 (2.2)	10 (1.6)
Oropharyngeal pain	3 (1.1)	3 (1.4)	5 (3.2)	8 (2.2)	11 (1.7)
Dizziness	1 (0.4)	0	5 (3.2)	5 (1.3)	6 (0.9)
**Aged ≥ 60 years, n (%)**	**Placebo (*n*** **= 69)**	**Erenumab 70 mg (*n*** **= 38)**	**Erenumab 140 mg (*n*** **= 35)**	**Erenumab 70 + 140 mg (*n*** **= 73)**	**Total (*N*** **= 142)**
**Number of participants with at least one AE**	41 (59.4)	15 (39.5)	17 (48.6)	32 (43.8)	73 (51.4)
Fatigue	2 (2.9)	2 (5.3)	2 (5.7)	4 (5.5)	6 (4.2)
Upper respiratory tract infection	2 (2.9)	3 (7.9)	1 (2.9)	4 (5.5)	6 (4.2)
Alopecia	0	0	2 (5.7)	2 (2.7)	2 (1.4)
Back pain	0	0	2 (5.7)	2 (2.7)	2 (1.4)
Injection-site pain	0	0	2 (5.7)	2 (2.7)	2 (1.4)
Ligament sprain	0	2 (5.3)	0	2 (2.7)	2 (1.4)
Musculoskeletal stiffness	0	2 (5.3)	0	2 (2.7)	2 (1.4)
Sinusitis	2 (2.9)	2 (5.3)	0	2 (2.7)	4 (2.8)
Viral upper respiratory tract infection	4 (5.8)	2 (5.3)	0	2 (2.7)	6 (4.2)

Data pooled from the phase 2 CM, STRIVE, ARISE, LIBERTY, and EMPOwER studies (safety analysis set). The summary shows TEAEs with onset day within the first 3 months (91 days) from the first administration of erenumab/placebo. Preferred terms are sorted in descending frequency of AEs in the 70/140 mg column and then alphabetically. A participant with multiple AEs is counted only once in the “at least one AE” row. A participant with multiple AEs with the same preferred term is counted only once for that preferred term

Abbreviations: *AEs* Adverse events, *CM* Chronic migraine, *TEAE* Treatment-emergent adverse event

Incidence of treatment-emergent SAEs by age group for both erenumab 70 mg and 140 mg doses was low: < 40 years, 0.9%; 40–49 years, 1.7%, and 50–59 years, 1.6%. No SAEs were reported in participants aged ≥ 60 years (Table 4). Most SAEs were reported in only one participant each across age group, except for migraine and uterine leiomyoma, which were reported in two participants in the 40- to 49-year age group and the 50- to 59-year age group, respectively.

**Table 4:** Treatment-emergent SAEs across age groups, by preferred term (safety analysis set)

**Aged < 40 years, n (%)**	**Placebo (*n*** **= 597)**	**Erenumab 70 mg (*n*** **= 514)**	**Erenumab 140 mg (*n*** **= 382)**	**Erenumab 70 + 140 mg (*n*** **= 896)**	**Total (*N*** **= 1493)**
**Number of participants with at least one SAE**	7 (1.2)	6 (1.2)	2 (0.5)	8 (0.9)	15 (1.0)
Asthenia	0	1 (0.2)	0	1 (0.1)	1 (0.1)
Cholelithiasis	0	1 (0.2)	0	1 (0.1)	1 (0.1)
Hypersensitivity	1 (0.2)	0	0	0	1 (0.1)
Gastroenteritis	0	1 (0.2)	0	1 (0.1)	1 (0.1)
Labyrinthitis	0	1 (0.2)	0	1 (0.1)	1 (0.1)
Urinary tract infection	0	1 (0.2)	0	1 (0.1)	1 (0.1)
Gastrointestinal infection	1 (0.2)	0	0	0	1 (0.1)
Parotitis	1 (0.2)	0	0	0	1 (0.1)
Viral infection	1 (0.2)	0	0	0	1 (0.1)
Cartilage injury	0	0	1 (0.3)	1 (0.1)	1 (0.1)
Flank pain	1 (0.2)	0	0	0	1 (0.1)
Intervertebral disc protrusion	1 (0.2)	0	0	0	1 (0.1)
Syncope	0	0	1 (0.3)	1 (0.1)	1 (0.1)
Migraine	1 (0.2)	0	0	0	1 (0.1)
Abortion	1 (0.2)	0	0	0	1 (0.1)
Ovarian cyst	0	1 (0.2)	0	1 (0.1)	1 (0.1)
**Aged 40–49 years, n (%)**	**Placebo (*n*** **= 421)**	**Erenumab 70 mg (*n*** **= 356)**	**Erenumab 140 mg (*n*** **= 275)**	**Erenumab 70 + 140 mg (*n*** **= 631)**	**Total (*N*** **= 1052)**
**Number of participants with at least one SAE**	8 (1.9)	6 (1.7)	5 (1.8)	11 (1.7)	19 (1.8)
Abdominal adhesions	0	0	1 (0.4)	1 (0.2)	1 (0.1)
Abdominal pain	0	0	1 (0.4)	1 (0.2)	1 (0.1)
Vomiting	1 (0.2)	0	0	0	1 (0.1)
Cholelithiasis	0	1 (0.3)	0	1 (0.2)	1 (0.1)
Cholecystitis	1 (0.2)	0	0	0	1 (0.1)
Cholecystitis acute	1 (0.2)	0	0	0	1 (0.1)
Hypersensitivity	1 (0.2)	0	0	0	1 (0.1)
Appendicitis	0	1 (0.3)	0	1 (0.2)	1 (0.1)
*Clostridium difficile* colitis	0	0	1 (0.4)	1 (0.2)	1 (0.1)
Kidney infection	0	0	1 (0.4)	1 (0.2)	1 (0.1)
Pyelonephritis	0	0	1 (0.4)	1 (0.2)	1 (0.1)
Sepsis	0	0	1 (0.4)	1 (0.2)	1 (0.1)
Urinary tract infection	1 (0.2)	0	0	0	1 (0.1)
Ankle fracture	0	0	1 (0.4)	1 (0.2)	1 (0.1)
Post-traumatic neck syndrome	0	1 (0.3)	0	1 (0.2)	1 (0.1)
Traumatic fracture	0	0	1 (0.4)	1 (0.2)	1 (0.1)
Fall	1 (0.2)	0	0	0	1 (0.1)
Costochondritis	0	1 (0.3)	0	1 (0.2)	1 (0.1)
Intervertebral disc protrusion	0	1 (0.3)	0	1 (0.2)	1 (0.1)
Migraine	0	1 (0.3)	1 (0.4)	2 (0.3)	2 (0.2)
Abortion	1 (0.2)	0	0	0	1 (0.1)
Endometriosis	1 (0.2)	0	0	0	1 (0.1)
**Aged 50**–**59 years, n (%)**	**Placebo (*n*** **= 262)**	**Erenumab 70 mg (*n*** **= 214)**	**Erenumab 140 mg (*n*** **= 158)**	**Erenumab 70 + 140 mg (*n*** **= 372)**	**Total (*N*** **= 634)**
**Number of participants with at least one SAE**	3 (1.1)	4 (1.9)	2 (1.3)	6 (1.6)	9 (1.4)
Pancreatitis	1 (0.4)	0	0	0	1 (0.2)
Non-cardiac chest pain	0	1 (0.5)	0	1 (0.3)	1 (0.2)
Vestibular neuronitis	0	0	1 (0.6)	1 (0.3)	1 (0.2)
Hyponatraemia	1 (0.4)	0	0	0	1 (0.2)
Back pain	0	1 (0.5)	0	1 (0.3)	1 (0.2)
Intervertebral disc protrusion	0	1 (0.5)	0	1 (0.3)	1 (0.2)
Fibroma	0	1 (0.5)	0	1 (0.3)	1 (0.2)
Uterine leiomyoma	1 (0.4)	0	1 (0.6)	1 (0.3)	2 (0.3)
Migraine	1 (0.4)	0	0	0	1 (0.2)
**Aged ≥ 60 years, n (%)**	**Placebo (*n*** **= 69)**	**Erenumab 70 mg (*n*** **= 38)**	**Erenumab 140 mg (*n*** **= 35)**	**Erenumab 70 + 140 mg (*n*** **= 73)**	**Total (*N*** **= 142)**
**Number of participants with at least one SAE**	0	0	0	0	0

Data pooled from phase 2 CM, STRIVE, ARISE, LIBERTY, and EMPOwER studies (safety analysis set). The summary contains TEAEs with onset day within the first 3 months (91 days) from the first administration of erenumab or placebo. A participant with multiple SAEs within a primary system organ class is counted only once in the total row. A participant with multiple occurrences of an SAE under one treatment is counted only once in this AE category for that treatment. System organ classes are presented in alphabetical order; preferred terms are sorted within system organ class in descending order of frequency in the 70/140 mg column, and then alphabetically. MedDRA Version which has been used for reporting is the same that was used in respective CSR analyses

Abbreviations: *AE* Adverse event, *CM* Chronic migraine, *SAE* Serious AE, *TEAE* Treatment-emergent AE

There was no difference in the incidence of AEs leading to study drug discontinuation between erenumab-treated (total) and placebo-treated groups by age (Table 5). In participants aged ≥ 60 years, one participant in the erenumab 140-mg group discontinued treatment due to nausea.

**Table 5:** TEAEs causing study drug discontinuation across age groups, by preferred term (safety analysis set)

**Aged < 40 years, n (%)**	**Placebo (*n*** **= 597)**	**Erenumab 70 mg (*n*** **= 514)**	**Erenumab 140 mg (*n*** **= 382)**	**Erenumab 70 + 140 mg (*n*** **= 896)**	**Total (*N*** **= 1493)**
**Number of participants with ≥ 1 AE causing study drug discontinuation**	5 (0.8)	6 (1.2)	4 (1.0)	10 (1.1)	15 (1.0)
Palpitations	0	1 (0.2)	0	1 (0.1)	1 (0.1)
Ventricular extrasystoles	0	0	1 (0.3)	1 (0.1)	1 (0.1)
Abdominal pain upper	0	1 (0.2)	0	1 (0.1)	1 (0.1)
Dyspepsia	0	0	1 (0.3)	1 (0.1)	1 (0.1)
Nausea	0	1 (0.2)	0	1 (0.1)	1 (0.1)
Vomiting	0	1 (0.2)	0	1 (0.1)	1 (0.1)
Injection-site pruritus	0	1 (0.2)	0	1 (0.1)	1 (0.1)
Injection-site rash	0	1 (0.2)	0	1 (0.1)	1 (0.1)
Hypersensitivity	1 (0.2)	0	0	0	1 (0.1)
Arthralgia	0	1 (0.2)	0	1 (0.1)	1 (0.1)
Pain in extremity	0	1 (0.2)	0	1 (0.1)	1 (0.1)
Dizziness	0	1 (0.2)	0	1 (0.1)	1 (0.1)
Headache	1 (0.2)	0	0	0	1 (0.1)
Pregnancy	1 (0.2)	1 (0.2)	0	1 (0.1)	2 (0.1)
Initial insomnia	0	1 (0.2)	0	1 (0.1)	1 (0.1)
Mood swings	0	0	1 (0.3)	1 (0.1)	1 (0.1)
Nervousness	0	1 (0.2)	0	1 (0.1)	1 (0.1)
Panic attack	1 (0.2)	0	0	0	1 (0.1)
Metrorrhagia	0	1 (0.2)	0	1 (0.1)	1 (0.1)
Rash maculo-papular	0	0	1 (0.3)	1 (0.1)	1 (0.1)
Alopecia	1 (0.2)	0	0	0	1 (0.1)
**Aged 40–49 years, n (%)**	**Placebo (*n*** **= 421)**	**Erenumab 70 mg (*n *= 356)**	**Erenumab 140 mg (*n*** **= 275)**	**Erenumab 70 + 140 mg (*n*** **= 631)**	**Total (*N*** **= 1052)**
**Number of participants with ≥ 1 AE causing study drug discontinuation**	3 (0.7)	5 (1.4)	3 (1.1)	8 (1.3)	11 (1.0)
Palpitations	0	0	1 (0.4)	1 (0.2)	1 (0.1)
Vertigo positional	0	1 (0.3)	0	1 (0.2)	1 (0.1)
Oral pain	0	0	1 (0.4)	1 (0.2)	1 (0.1)
Fatigue	1 (0.2)	0	1 (0.4)	1 (0.2)	2 (0.2)
Allergy to arthropod sting	0	1 (0.3)	0	1 (0.2)	1 (0.1)
Pain in extremity	1 (0.2)	0	0	0	1 (0.1)
Hypoaesthesia	1 (0.2)	0	0	0	1 (0.1)
Affect lability	0	1 (0.3)	0	1 (0.2)	1 (0.1)
Metrorrhagia	0	0	1 (0.4)	1 (0.2)	1 (0.1)
Mechanical urticaria	0	1 (0.3)	0	1 (0.2)	1 (0.1)
**Aged 50–59 years, n (%)**	**Placebo (*n*** **= 262)**	**Erenumab 70 mg (*n*** **= 214)**	**Erenumab 140 mg (*n*** **= 158)**	**Erenumab 70 + 140 mg (*n*** **= 372)**	**Total (*N*** **= 634)**
**Number of participants with ≥ 1 AE causing study drug discontinuation**	2 (0.8)	2 (0.9)	3 (1.9)	5 (1.3)	7 (1.1)
Vertigo positional	0	0	1 (0.6)	1 (0.3)	1 (0.2)
Constipation	0	0	1 (0.6)	1 (0.3)	1 (0.2)
Gastrooesophageal reflux disease	1 (0.4)	0	0	0	1 (0.2)
Fatigue	0	1 (0.5)	0	1 (0.3)	1 (0.2)
Vestibular neuronitis	0	0	1 (0.6)	1 (0.3)	1 (0.2)
Arthralgia	0	1 (0.5)	0	1 (0.3)	1 (0.2)
Cough	1 (0.4)	0	0	0	1 (0.2)
Dyspnoea	1 (0.4)	0	0	0	1 (0.2)
Alopecia	1 (0.4)	0	0	0	1 (0.2)
**Aged ≥ 60 years, n (%)**	**Placebo (*n*** **= 69)**	**Erenumab 70 mg (*n*** **= 38)**	**Erenumab 140 mg (*n*** **= 35)**	**Erenumab 70 + 140 mg (*n*** **= 73)**	**Total (*N*** **= 142)**
**Number of participants with ≥ 1 AE causing study drug discontinuation**	1 (1.4)	0	1 (2.9)	1 (1.4)	2 (1.4)
Nausea	0	0	1 (2.9)	1 (1.4)	1 (0.7)
Erythema	1 (1.4)	0	0	0	1 (0.7)

Data pooled from phase 2 CM, STRIVE, ARISE, LIBERTY, and EMPOwER studies (safety analysis set). The summary contains TEAEs with onset day within the first 3 months (91 days) from the first administration of erenumab or placebo. A participant with multiple AEs within a primary system organ class is counted only once in the total row. A participant with multiple occurrences of an AE under one treatment is counted only once in this AE category for that treatment. Preferred terms are sorted in descending order of frequency in the 70/140 mg column, and then alphabetically

Abbreviations: *AE* Adverse event, *CM* Chronic migraine, *SAE* Serious AE, *TEAE* Treatment-emergent AE

There were no notable imbalances among treatment groups in AEs, SAEs, or severity of AEs by age group during the double-blind treatment phase. Incidence of CV and cerebrovascular AEs were unremarkable across the age groups (Table 6). Notably, no CV and cerebrovascular AESIs were reported in the erenumab 140-mg group in participants aged ≥ 60 years, possibly due to the relatively lower number of participants receiving erenumab 140 mg as compared to erenumab 70 mg or placebo.

**Table 6:** Summary of cardiovascular and cerebrovascular AEs by age groups (safety analysis set) by preferred term

**Aged < 40 years, n (%)**	**Placebo (*n*** **= 597)**	**Erenumab 70 mg (*n*** **= 514)**	**Erenumab 140 mg (*n*** **= 382)**	**Erenumab 70 + 140 mg (*n*** **= 896)**	**Total (*N*** **= 1493)**
**Participants with CV/ cerebrovascular TEAEs**	12 (2.0)	7 (1.4)	10 (2.6)	17 (1.9)	29 (2.0)
Palpitations	2 (0.3)	2 (0.4)	3 (0.8)	5 (0.6)	7 (0.5)
Hypertension	2 (0.3)	3 (0.6)	0	3 (0.3)	5 (0.3)
Hot flush	2 (0.3)	0	2 (0.5)	2 (0.2)	4 (0.3)
Flushing	1 (0.2)	0	1 (0.3)	1 (0.1)	2 (0.1)
Bundle branch block right	0	1 (0.2)	0	1 (0.1)	1 (0.1)
Cardiac flutter	0	0	1 (0.3)	1 (0.1)	1 (0.1)
Diastolic hypertension	0	0	1 (0.3)	1 (0.1)	1 (0.1)
Haematoma	1 (0.2)	0	0	0	1 (0.1)
Hypotension	1 (0.2)	0	0	0	1 (0.1)
Junctional ectopic tachycardia	0	1 (0.2)	0	1 (0.1)	1 (0.1)
Orthostatic hypotension	0	0	1 (0.3)	1 (0.1)	1 (0.1)
Sinus tachycardia	1 (0.2)	0	0	0	1 (0.1)
Supraventricular extrasystoles	0	0	1 (0.3)	1 (0.1)	1 (0.1)
Tachycardia	1 (0.2)	0	0	0	1 (0.1)
Tricuspid valve incompetence	1 (0.2)	0	0	0	1 (0.1)
Ventricular extrasystoles	0	0	1 (0.3)	1 (0.1)	1 (0.1)
**Aged 40–49 years, n (%)**	**Placebo (*n*** **= 421)**	**Erenumab 70 mg (*n*** **= 356)**	**Erenumab 140 mg (*n*** **= 275)**	**Erenumab 70 + 140 mg (*n*** **= 631)**	**Total (*N*** **= 1052)**
**Participants with CV/ cerebrovascular TEAEs**	10 (2.4)	5 (1.4)	7 (2.5)	12 (1.9)	22 (2.1)
Hypertension	2 (0.5)	2 (0.6)	1 (0.4)	3 (0.5)	5 (0.5)
Palpitations	2 (0.5)	1 (0.3)	2 (0.7)	3 (0.5)	5 (0.5)
Extrasystoles	1 (0.2)	0	1 (0.4)	1 (0.2)	2 (0.2)
Hot flush	2 (0.5)	0	0	0	2 (0.2)
Arrhythmia	1 (0.2)	0	0	0	1 (0.1)
Capillary fragility	0	0	1 (0.4)	1 (0.2)	1 (0.1)
Deep vein thrombosis	0	1 (0.3)	0	1 (0.2)	1 (0.1)
Haematoma	1 (0.2)	0	0	0	1 (0.1)
Hypertensive crisis	0	1 (0.3)	0	1 (0.2)	1 (0.1)
Hypotension	0	0	1 (0.4)	1 (0.2)	1 (0.1)
Supraventricular tachycardia	1 (0.2)	0	0	0	1 (0.1)
Thrombosis	0	0	1 (0.4)	1 (0.2)	1 (0.1)
**Aged 50**–**59 years, n (%)**	**Placebo (*n*** **= 262)**	**Erenumab 70 mg (*n*** **= 214)**	**Erenumab 140 mg (*n*** **= 158)**	**Erenumab 70 + 140 mg (*n*** **= 372)**	**Total (*N*** **= 634)**
**Participants with CV/ cerebrovascular TEAEs**	7 (2.7)	4 (1.9)	4 (2.5)	8 (2.2)	1 (0.2)
Hot flush	1 (0.4)	1 (0.5)	2 (1.3)	3 (0.8)	5 (0.8)
Flushing	0	1 (0.5)	0	1 (0.3)	4 (0.6)
Hypertension	4 (1.5)	1 (0.5)	0	1 (0.3)	2 (0.3)
Atrioventricular block first degree	0	1 (0.5)	0	1 (0.3)	1 (0.2)
Palpitations	1 (0.4)	0	1 (0.6)	1 (0.3)	1 (0.2)
Peripheral coldness	0	0	1 (0.6)	1 (0.3)	1 (0.2)
**Aged ≥ 60 years, n (%)**	**Placebo (*n*** **= 69)**	**Erenumab 70 mg (*n*** **= 38)**	**Erenumab 140 mg (*n*** **= 35)**	**Erenumab 70 + 140 mg (*n*** **= 73)**	**Total (*N*** **= 142)**
**Participants with CV/ cerebrovascular TEAEs**	4 (5.8)	2 (5.3)	0	2 (2.7)	6 (4.2)
Hot flush	1 (1.4)	1 (2.6)	0	1 (1.4)	2 (1.4)
Flushing	1 (1.4)	0	0	0	1 (0.7)
Hypertensive crisis	0	1 (2.6)	0	1 (1.4)	1 (0.7)
Tachycardia	1 (1.4)	0	0	0	1 (0.7)
Ventricular extrasystoles	1 (1.4)	0	0	0	1 (0.7)

Data pooled from phase 2 CM, STRIVE, ARISE, LIBERTY, and EMPOwER studies (safety analysis set). The table contains AEs with onset date within 91 days from the first administration of erenumab. % = n/N × 100

The table contains AEs with onset date within 91 days from the first administration of the study drug. AEs are presented at the dose level at which the event occurred, so individuals who received erenumab at more than 1 dose level during the study were counted in both dose levels. Therefore, the total erenumab column may not be the sum of the individuals included in each of the individual dose levels

Abbreviations: *AE* Adverse event, *CM* Chronic migraine, *CV* Cardiovascular, *N* Number of individuals exposed to the given dose level, *n* number of individuals reporting at least 1 occurrence of an AE in that class within 91 days from the first administration, *SAE* Serious AE, *TEAE* Treatment-emergent AE

The incidence of GI AEs across age groups and treatment groups was low, with an overall incidence of 290 (8.7%). The only remarkable GI AE commonly found across the age groups was constipation (Table 7). Incidence of constipation was higher in the erenumab 140 mg group compared with erenumab 70 mg group (below 4% across age groups for both erenumab doses), and lowest in those receiving placebo across all age groups. Participants aged ≥ 60 years also reported other GI AEs including nausea, dyspepsia, and toothache. Incidence of nausea was similar across the treatment groups.

**Table 7:** Summary of GI AEs across age groups with incidence of ≥ 2.5% in any erenumab group by preferred term (safety analysis set)

**Aged < 40 years, n (%)**	**Placebo (*n*** **= 597)**	**Erenumab 70 mg (*n*** **= 514)**	**Erenumab 140 mg (*n*** **= 382)**	**Erenumab 70 + 140 mg (*n*** **= 896)**	**Total (***N* **= 1493)**
**Participants with GI TEAEs**	47 (7.9)	48 (9.3)	44 (11.5)	92 (10.3)	139 (9.3)
Constipation	6 (1.0)	16 (3.1)	15 (3.9)	31 (3.5)	37 (2.5)
**Aged 40–49 years, n (%)**	**Placebo (*n*** **= 421)**	**Erenumab 70 mg (*n*** **= 356)**	**Erenumab 140 mg (*n*** **= 275)**	**Erenumab 70 + 140 mg (*n*** **= 631)**	**Total (*N*** **= 1052)**
**Participants with GI TEAEs**	37 (8.8)	26 (7.3)	19 (6.9)	45 (7.1)	82 (7.8)
Constipation	7 (1.7)	8 (2.2)	7 (2.5)	15 (2.4)	22 (2.1)
**Aged 50**–**59 years, n (%)**	**Placebo (*n*** **= 262)**	**Erenumab 70 mg (*n*** **= 214)**	**Erenumab 140 mg (*n*** **= 158)**	**Erenumab 70 + 140 mg (*n*** **= 372)**	**Total (*N*** **= 634)**
**Participants with GI TEAEs**	25 (9.5)	17 (7.9)	15 (9.5)	32 (8.6)	57 (9.0)
Constipation	3 (1.1)	4 (1.9)	7 (4.4)	11 (3.0)	14 (2.2)
**Aged ≥ 60 years, n (%)**	**Placebo (*n*** **= 69)**	**Erenumab 70 mg (*n*** **= 38)**	**Erenumab 140 mg (*n*** **= 35)**	**Erenumab 70 + 140 mg (*n*** **= 73)**	**Total (*N*** **= 142)**
**Participants with GI TEAEs**	7 (10.1)	3 (7.9)	2 (5.7)	5 (6.8)	12 (8.5)
Constipation	1 (1.4)	0	1 (2.9)	1 (1.4)	2 (1.4)
Dyspepsia	0	1 (2.6)	0	1 (1.4)	1 (0.7)
Nausea	2 (2.9)	1 (2.6)	1 (2.9)	2 (2.7)	4 (2.8)
Toothache	1 (1.4)	1 (2.6)	0 (0)	1 (1.4)	2 (1.4)

Data pooled from phase 2 CM, STRIVE, ARISE, LIBERTY, and EMPOwER studies (safety analysis set). The table contains AEs with onset date within 91 days from the first administration of the study drug. AEs are presented at the dose level at which the event occurred, so individuals who received erenumab at more than 1 dose level during the study were counted in both dose levels. Therefore, the total erenumab column may not be the sum of the individuals included in each of the individual dose levels. % = n/N × 100

Abbreviations: *AE* Adverse event, *CM* Chronic migraine, *GI* Gastrointestinal, *N* Number of individuals exposed to the given dose level, *n* number of individuals reporting at least 1 occurrence of an AE in that class within 91 days from the first administration, *TEAE* Treatment-emergent adverse event

The incidence of CV, cerebrovascular events, and constipation were comparable for both erenumab treatment groups and placebo within each age subgroup (Tables 6 and 7). Similar trends were seen across other age groups except for individuals aged ≥ 60 years who presented with a higher percent of CV and cerebrovascular events (4.2%) (Table 6).

## Discussion

This pooled analysis demonstrated that erenumab treatment (70 mg and 140 mg) showed similar incidence rates of TEAEs versus placebo across the age groups evaluated. Treatment with erenumab in patients with EM and CM also showed favorable CV and cerebrovascular safety in all age groups. Only minimal GI side effects were observed, even in older patients (≥ 60 years), suggesting that erenumab is a suitable preventive treatment option across all evaluated age groups. Importantly, because erenumab is a mAb, it is not expected to have drug-drug interactions, which are often a secondary challenge in older patients [22].

Management of migraine in older individuals can be complex due to the presence of multiple medical comorbidities, such as diabetes, hypertension, heart disease, and other cerebrovascular events, and their associated medications [23]. Additionally, older individuals are often faced with age-related physiological changes, such as slowing of gastric emptying, and an altered drug metabolism rate [24]. The long-term use of medications for chronic conditions like diabetes, hypertension, thyroid, and arthritis may exacerbate migraine symptoms or trigger migraine, thereby stressing the special attention that is needed when developing treatment plans in these individuals [6, 25].

With an increase in the global life expectancy, there is a shift towards finding optimal solutions to manage health problems in an aging population. Migraine already has a significant impact in older individuals and this impact is likely to increase in line with aging population demographics. At the present time, most standard oral preventive treatments should be used with caution in older individuals due to their unfavorable safety and tolerability profile. This pooled analysis shows that erenumab is a suitable, well-tolerated and effective treatment alternative across all age groups studied.

Older individuals receiving medications can be at a higher risk of CV and cerebrovascular AEs, compounded by pre-existing medical conditions. Furthermore, CGRP is a potent vasodilator and plays an important role in regulating vascular resistance and regional organ blood flow [26]; therefore, erenumab, as a CGRP receptor antagonist, could in theory increase the overall cardiovascular risk profile in all age groups. Indeed, development of hypertension and worsening of preexisting hypertension has been reported following the use of erenumab in a post-marketing setting, [27] leading to a recent revision of the USPI for erenumab to include hypertension as a warning and an adverse drug reaction [22]. However, in this pooled analysis, the incidence of TEAEs of hypertension was low, and our findings suggest that erenumab is well tolerated and poses no additional CV and cerebrovascular risk in patients of all age groups, including older individuals. Similarly, the incidence of TEAEs of constipation, another AE that is frequently reported in a post-marketing setting following use of erenumab, [22, 27, 28] was low across all age groups and comparable across treatment arms in this study. Only one patient in the 50- to 59-year age group receiving 140 mg erenumab discontinued treatment due to constipation. Together, these findings add to the growing body of evidence supporting the safety and tolerability of erenumab across age groups and migraine types.

Kudrow et al. have assessed the vascular safety profile of erenumab across four large placebo-controlled trials with similar findings to this pooled analysis [19]. The CV, cerebrovascular, and peripheral vascular safety profile of erenumab was comparable to that of placebo, with no CV risk associated with erenumab use. Another pooled safety analysis of erenumab that included four double-blind, randomized, placebo-controlled trials and their open-label extensions for > 3 years, assessed the longest-term integrated safety data [20]. A favorable safety and tolerability profile was determined for erenumab, supporting its use as a chronic treatment for migraine prevention. However, it should be acknowledged that these randomized-controlled trials had strict inclusion criteria and thus findings cannot be generalized to the general population. We must continue to rely on post-marketing surveillance and real-world observations to provide new insights into the safety of erenumab in a wider patient population [27, 29, 30].

Recently, other pooled analyses of large, placebo-controlled trials have evaluated the comprehensive safety and tolerability profile of two CGRP inhibitors, eptinezumab [31] and fremanezumab [32], across a broad spectrum of participants with EM or CM. Notably, pooled subgroup analysis assessing the safety profile of fremanezumab in participants aged ≥ 60 years reported comparable findings with the overall pooled population [33]. Furthermore, the CONQUER study, which enrolled patients aged 18‒75 years, found no clinically significant safety findings for AEs, laboratory analytes, vital signs, or ECGs in the subgroup of patients aged 65‒75 years treated with galcanezumab, albeit the sample size was low (*N* = 29) [34]. While this provides preliminary evidence that use of CGRP mAbs may be safe in individuals beyond 65 years, real-life studies in larger populations of older individuals are needed.

### Strengths

This is the first pooled analysis to compare the safety and tolerability of erenumab (70 mg and 140 mg vs placebo) in individuals with EM and CM across different age groups enrolling participants across different global regions. Despite the stringent exclusion criteria, the pooled analysis had nearly 150 individuals in the 60- to 66-year age group.

### Limitations

Due to ethical considerations, there were several key exclusion criteria for participants with a history of CV disease, etc., which is a major limitation of this analysis as it focused heavily on hypertension and CV outcomes. Also, the trials included in this pooled analysis had a cut-off age of 65 years. As a result, the ≥ 60 year age group had a relatively small sample size compared with other age groups. Thus, any variability between treatments in the ≥ 60 years age group may be a result of the small sample size. The incidence of laboratory parameters, ECG or vital signs abnormalities were not analyzed here. There were analyzed only if reported as AEs (if deemed clinically significant), thus the safety perspective is not complete. Safety data from the 12-week DBTP of each study were included in this pooled analysis, which may be a relatively short period to assess safety. It is suggested that the long-term safety may be evaluated using the longer open-label period of the studies. Finally, the analysis was performed without analyzing the statistical significance of the safety trends.

## Conclusions

A comprehensive understanding of the safety profile of erenumab is of key importance to treatment planning for effective migraine prevention, particularly in individuals aged ≥ 50 years who are at higher risk of incurring medication-related side effects. This pooled analysis established that the overall safety profile of erenumab 70 mg and 140 mg was comparable with placebo across the age groups evaluated in 3345 individuals with EM or CM, with no increase in AEs in those aged ≥ 50 years. That no notable safety events were found in older individuals receiving erenumab when compared with the other age groups is of key importance and suggests that erenumab is a safe and well-tolerated treatment option for older patients.

## Supplementary Information

**Additional file 1:** **Supplementary Table 1.** Baseline characteristics of pooled studies. **Supplementary Appendix 1.**List of Independent Ethics Committees (IEC) or Institutional Review Boards(IRB) by study center.

## Data Availability

The datasets described in this report are available by request from the corresponding author or Novartis Pharma AG. De-identified participant data are not available for legal and ethical reasons.

## Abbreviations

AE: Adverse event
BMI: Body mass index
CGRP: Calcitonin gene-related peptide
CM: Chronic migraine
CV: Cardiovascular
CTCAE: Common Terminology Criteria for Adverse Events
DBTP: Double-blind treatment phase
EM: Episodic migraine
IP: Investigational product
IV: Intravenous
MedDRA: Medical Dictionary for Regulatory Activities
NA: Not applicable
QoL: Quality of life
SAE: Serious adverse event
SD: Standard deviation
TEAE: Treatment-emergent adverse event
