# Supplementary material for: Safety and tolerability of erenumab in individuals with episodic or chronic migraine across age groups: a pooled analysis of placebo-controlled trials
Source: J Headache Pain. 2022 Aug 18;23(1):104. doi: 10.1186/s10194-022-01470-4 (PMC9386939; doi:10.1186/s10194-022-01470-4)
Supplement: Supplementary file 1 — Additional file 1: Supplementary Table 1. Baseline characteristics of pooled studies. Supplementary Appendix 1.List of Independent Ethics Committees (IEC) or Institutional Review Boards(IRB) by study center. [file 10194_2022_1470_MOESM1_ESM.docx]

# **Supplementary Table 1. Baseline characteristics of pooled studies**

| **Characteristic** | **Placebo N=1359** | **Erenumab 70 mg N=1132** | **Erenumab 140 mg N=854** | **All patients N=3345** |
| --- | --- | --- | --- | --- |
| **Age**, mean (SD)* | 41.1 (11.1) | 40.3 (11.1) | 40.7 (10.9) | 40.7 (11.1) |
| **Female**, n (%) | 1131 (83.2) | 951 (84.0) | 713 (83.5) | 2795 (83.6) |
| **Race**, n (%) |  |  |  |  |
| **Asian** | 277 (20.4) | 280 (24.7) | 180 (21.1) | 737 (22.0) |
| **White** | 974 (71.7) | 766 (67.7) | 616 (72.1) | 2356 (70.4) |
| **BMI** (kg/m^2^), mean (SD) | 26.2 (5.6) | 26.2 (5.6) | 25.9 (5.4) | 26.1 (5.6) |
| **MMDs,** mean (SD) | 10.5 (5.1) | 9.8 (4.7) | 10.5 (5.1) | 10.3 (5.0) |
| **MMDs, categories**,  n (%) |  |  |  |  |
| 8–14 Migraine days per month | 656 (48.3) | 544 (48.1) | 411 (48.1) | 1611 (48.2) |
| ≥15 Migraine days per month | 224 (16.5) | 149 (13.2) | 142 (16.6) | 515 (15.4) |
| **Medication overuse**, n (%)** |  |  |  |  |
| Yes | 117 (8.6) | 79 (7.0) | 78 (9.1) | 274 (8.2) |
| No | 169 (12.4) | 112 (9.9) | 112 (13.1) | 393 (11.7) |
| **Prior migraine prophylactic failures**, n (%) |  |  |  |  |
| 0 | 686 (50.5) | 655 (57.9) | 414 (48.5) | 1755 (52.5) |
| 1 | 258 (19.0) | 224 (19.8) | 131 (15.3) | 613 (18.3) |
| 2 | 168 (12.4) | 120 (10.6) | 133 (15.6) | 421 (12.6) |
| ≥3 | 247 (18.2) | 133 (11.7) | 176 (20.6) | 556 (16.6) |
| **Framingham risk factors** | | | | |
| **Cigarette** **use**, n (%) |  |  |  |  |
| Current | 64 (4.7) | 67 (5.9) | 41 (4.8) | 172 (5.1) |
| Former | 115 (8.5) | 95 (8.4) | 51 (6.0) | 261 (7.8) |
| Never | 431 (31.7) | 439 (38.8) | 227 (26.6) | 1097 (32.8) |
| Unknown | 749 (55.1) | 531 (46.9) | 535 (62.6) | 1815 (54.3) |
| **Diabetes,** n (%) | 26 (1.9) | 20 (1.8) | 10 (1.2) | 56 (1.7) |
| **Hypertension**, n (%) | 169 (12.4) | 109 (9.6) | 87 (10.2) | 365 (10.9) |
| **Total** **cholesterol** (mmol/L), mean (SD) | 5.0 (1.0) | 5.0 (0.9) | 5.0 (1.0) | 4.9 (1.0) |
| **HDL cholesterol** (mmol/L), mean (SD) | 1.5 (0.4) | 1.5 (0.4) | 1.5 (0.4) | 1.5 (0.4) |
| **Systolic blood pressure (mmHg),** mean (SD) | 119 (12.9) | 118 (13.0) | 119 (13.0) | 119 (12.9) |
| **Coronary artery disease**, n (%) | 3 (0.2) | 1 (0.1) | 2 (0.2) | 6 (0.2) |
| **Cerebrovascular or peripheral artery disease**, n (%) | 8 (0.6) | 3 (0.3) | 6 (0.7) | 17 (0.5) |

Data pooled from phase 2 CM study (randomized analysis set), STRIVE study (full analysis set), ARISE study (full analysis set), LIBERTY study (randomized analysis set), EMPOwER (randomized analysis set) and presented as mean (SD), unless stated. *Although the inclusion criteria in all studies comprised individuals aged between 18 to 65 years, there was one participant aged 17 years and one aged 66 years in the data pool. **Medication overuse was captured only for phase 2 CM study. Smoking-related information was not collected in LIBERTY and EMPOwER studies. Hence, these were counted under "Unknown" category in the parameter "Cigarette use". Total cholesterol and HDL-related information was not collected in LIBERTY study.

Abbreviations: BMI, body mass index; CM, chronic migraine; HDL, high-density lipoprotein; MMD, monthly migraine days; SD, standard deviation.

# **Supplementary Appendix 1**

## **List of Independent Ethics Committees (IEC) or Institutional Review Boards (IRB) by study center**

### **Phase 2 chronic migraine study (ClinicalTrials.gov identifier: NCT02066415)**

| **Site no.** | **Site name** | **Ethics Committee or  Institutional Review Board** | **City, State/Province, Postal Code Country** |
| --- | --- | --- | --- |
| 16001 | Calgary Headache Assessment and Management Program | University of Calgary Conjoint Health Research Ethics Board | 2500 University Drive Northwest, 3rd Floor MacKimmie Library Tower MLT 300, Calgary, AB, T2N 1N4, Canada |
| 16004 | Centre Hospitalier de L'Universite de Montreal Notre‐Dame | Comite d'ethique de la recherche du Centre Hospitalier de l'Universite de Montreal (CHUM) | 900 rue St‐Denis, Pavillon R, 3rd Floor, Montreal, QC, H2X 0A9, Canada |
| 21001 | Dado Medical sro | Eticka komise pri IKEM a TN | Videnska 800, Praha 4, 140 59, Czech Republic |
| 21001 | Dado Medical sro | Eticka komise Fakultni Nemocnice u sv Anny v Brne | Pekarska 53, Brno, 656 91, Czech Republic |
| 21002 | Thomayerova nemocnice | Eticka komise pri IKEM a TN | Videnska 800, Praha 4, 140 59, Czech Republic |
| 21002 | Thomayerova nemocnice | Eticka komise Fakultni Nemocnice u sv Anny v Brne | Pekarska 53, Brno, 656 91, Czech Republic |
| 21003 | Fakultni nemocnice u svate Anny v Brne | Eticka komise Fakultni Nemocnice u sv Anny v Brne | Pekarska 53, Brno, 656 91, Czech Republic |
| 21003 | Fakultni nemocnice u svate Anny v Brne | Eticka komise Fakultni Nemocnice u sv Anny v Brne | Pekarska 53, Brno, 656 91, Czech Republic |
| 21005 | Quattromedica | Eticka komise Fakultni Nemocnice u sv Anny v Brne | Pekarska 53, Brno, 656 91, Czech Republic |
| 21005 | Quattromedica | Eticka komise Fakultni Nemocnice u sv Anny v Brne | Pekarska 53, Brno, 656 91, Czech Republic |
| 22001 | Glostrup Hospital | Den Videnskabsetiske Komité for RegionHovedstaden | Regionsgården Kongens Vænge 2, Hillerød, 3400,Denmark |
| 24001 | Helsingin Paansarkykeskus Aava | HUS Ethics Committee (HUS Tutkimuseettiset toimikunnat) | Tukholmankatu 8 C, Biomedicum Helsinki 2C, PL 705,HUS, 00029, Finland |
| 24003 | Terveystalo Oulu, Diapolis | HUS Ethics Committee (HUS Tutkimuseettiset toimikunnat) | Tukholmankatu 8 C, Biomedicum Helsinki 2C, PL 705,HUS, 00029, Finland |
| 24004 | Terveystalo Tampere | HUS Ethics Committee (HUS Tutkimuseettiset toimikunnat) | Tukholmankatu 8 C, Biomedicum Helsinki 2C, PL 705,HUS, 00029, Finland |
| 24005 | Terveystalo Pulssi | HUS Ethics Committee (HUS Tutkimuseettiset toimikunnat) | Tukholmankatu 8 C, Biomedicum Helsinki 2C, PL 705,HUS, 00029, Finland |
| 26001 | Universitätsklinikum Hamburg‐Eppendorf | Geschäftstelle der Ethikkommission beim Landesamt für Gesundheit | Fehrbelliner Platz 1, Berlin, 10707, Germany |
| 26002 | Charité Campus Mitte | Geschäftstelle der Ethikkommission beim Landesamt für Gesundheit | Fehrbelliner Platz 1, Berlin, 10707, Germany |
| 26003 | Praxis Dr Stude | Geschäftstelle der Ethikkommission beim Landesamt für Gesundheit | Fehrbelliner Platz 1, Berlin, 10707, Germany |
| 26005 | Schmerzzentrum Berlin GmbH | Geschäftstelle der Ethikkommission beimLandesamt für Gesundheit | Fehrbelliner Platz 1, Berlin, 10707, Germany |
| 26006 | Migräne‐ und Kopfschmerzklinik Königstein | Geschäftstelle der Ethikkommission beim Landesamt für Gesundheit | Fehrbelliner Platz 1, Berlin, 10707, Germany |
| 26007 | Universitätsklinikum Essen | Geschäftstelle der Ethikkommission beim Landesamt für Gesundheit | Fehrbelliner Platz 1, Berlin, 10707, Germany |
| 26014 | Schmerzklinik Kiel | Geschäftstelle der Ethikkommission beimLandesamt für Gesundheit | Fehrbelliner Platz 1, Berlin, 10707, Germany |
| 48001 | Centrum Leczenia Padaczki i Migreny w Krakowie | Komisja Bioetyczna przy Okregowej Izbie Lekarskiej w Lodzi | ulica Czerwona 3, Lodz, 93‐005, Poland |
| 48005 | Gabinet Neurologiczny Prof Adam Stepien | Komisja Bioetyczna przy Okregowej Izbie Lekarskiej w Lodzi | ulica Czerwona 3, Lodz, 93‐005, Poland |
| 59003 | Stockholm Neurocenter | Regionala Etikprövningsnämnden i Stockholm | Tomtebodavägen 18A, Widerströmska huset, Plan 3, Solna, 17165, Sweden |
| 59005 | S3 Clinical Research Centers | Regionala Etikprövningsnämnden i Stockholm | Tomtebodavägen 18A, Widerströmska huset, Plan 3, Solna, 17165, Sweden |
| 59006 | Smartkliniken St Olof | Regionala Etikprövningsnämnden i Stockholm | Tomtebodavägen 18A, Widerströmska huset, Plan 3, Solna, 17165, Sweden |
| 59007 | Neurology Clinic | Regionala Etikprövningsnämnden i Stockholm | Tomtebodavägen 18A, Widerströmska huset, Plan 3, Solna, 17165, Sweden |
| 59009 | Karolinska Universitetssjukhuset, Huddinge | Regionala Etikprövningsnämnden i Stockholm | Tomtebodavägen 18A, Widerströmska huset, Plan 3, Solna, 17165, Sweden |
| 65001 | Kings College Hospital | Quintiles Laboratories | Rosebank, The Alba Centre, Quintiles Laboratories, Livingston, EH54 7EG, United Kingdom |
| 65001 | Kings College Hospital | London – City and East Research Ethics Committee | Lewins Mead, Whitefriars, Level 3, Block B, London – City and East Research Ethics Committee, Bristol, BS1 2NT, United Kingdom |
| 65002 | Royal Stoke University Hospital | Quintiles Laboratories | Rosebank, The Alba Centre, Quintiles Laboratories, Livingston, EH54 7EG, United Kingdom |
| 65002 | Royal Stoke University Hospital | London – City and East Research Ethics Committee | Lewins Mead, Whitefriars, Level 3, Block B, London – City and East Research Ethics Committee, Bristol, BS1 2NT, United Kingdom |
| 65003 | Hull Royal Infirmary | Quintiles Laboratories | Rosebank, The Alba Centre, Quintiles Laboratories, Livingston, EH54 7EG, United Kingdom |
| 65003 | Hull Royal Infirmary | London – City and East Research Ethics Committee | Lewins Mead, Whitefriars, Level 3, Block B, London – City and East Research Ethics Committee, Bristol, BS1 2NT, United Kingdom |
| 65004 | Queen Elizabeth University Hospital | Quintiles Laboratories | Rosebank, The Alba Centre, Quintiles Laboratories, Livingston, EH54 7EG, United Kingdom |
| 65004 | Queen Elizabeth University Hospital | London – City and East Research Ethics Committee | Lewins Mead, Whitefriars, Level 3, Block B, London – City and East Research Ethics Committee, Bristol, BS1 2NT, United Kingdom |
| 66001 | Stanford Healthcare | Stanford University Administrative Panel on Human Subjects in Medical Research | 3000 El Camino Real, Five Palo Alto Square 4th Floor, Palo Alto, CA, 94306, USA |
| 66002 | Mercy Health Research | Mercy Hospital Saint Louis | 615 South New Ballas Road, St Louis, MO, 63141, USA |
| 66002 | Mercy Health Research | Schulman Associates IRB | 4445 Lake Forest Drive, Suite 300, Cincinnati, OH, 45242, USA |
| 66003 | Nashville Neuroscience Group | Schulman Associates IRB | 4445 Lake Forest Drive, Suite 300, Cincinnati, OH, 45242, USA |
| 66004 | Clinvest Research LLC | Schulman Associates IRB | 4445 Lake Forest Drive, Suite 300, Cincinnati, OH, 45242, USA |
| 66005 | Scripps Clinic, Division of Neurology | Scripps Office for the Protection of Research Subjects | 11025 North Torrey Pines Road, Suite 200, La Jolla, CA, 92037, USA |
| 66006 | Headache Wellness Center | Schulman Associates IRB | 4445 Lake Forest Drive, Suite 300, Cincinnati, OH, 45242, USA |
| 66007 | Comprehensive Headache Center | Baylor Research Institute | 3310 Live Oak Street, Suite 501, Dallas, TX, 75204, USA |
| 66008 | Jerome Goldstein, San Francisco Clinical Research Center | Schulman Associates IRB | 4445 Lake Forest Drive, Suite 300, Cincinnati, OH, 45242, USA |
| 66010 | Tidewater Integrated Medical Research | Schulman Associates IRB | 4445 Lake Forest Drive, Suite 300, Cincinnati, OH, 45242, USA |
| 66012 | Texas Neurology, PA | Schulman Associates IRB | 4445 Lake Forest Drive, Suite 300, Cincinnati, OH, 45242, USA |
| 66015 | University of Washington Medical Center | Western Institutional Review Board | 1019 39th Avenue Southeast, Suite 120, Puyallup, WA, 98374, USA |
| 66016 | New England Regional Headache Center, Inc. | Schulman Associates IRB | 4445 Lake Forest Drive, Suite 300, Cincinnati, OH, 45242, USA |
| 66017 | New England Institute for Clinical Research | Schulman Associates IRB | 4445 Lake Forest Drive, Suite 300, Cincinnati, OH, 45242, USA |
| 66018 | Neurostudies Net LLC | Schulman Associates IRB | 4445 Lake Forest Drive, Suite 300, Cincinnati, OH, 45242, USA |
| 66020 | Newport Beach Clinical Research Associates | Schulman Associates IRB | 4445 Lake Forest Drive, Suite 300, Cincinnati, OH, 45242, USA |
| 66021 | Scott and White Memorial Hospital and Clinic | Scott and White Institutional Review Board | 2401 South 31st Street, Temple, TX, 76508, USA |
| 66022 | Mid Atlantic Headache Institute | Schulman Associates IRB | 4445 Lake Forest Drive, Suite 300, Cincinnati, OH, 45242, USA |
| 66023 | Renown Institute for Neurosciences | Schulman Associates IRB | 4445 Lake Forest Drive, Suite 300, Cincinnati, OH, 45242, USA |
| 66024 | Westside Family Medical Center | Schulman Associates IRB | 4445 Lake Forest Drive, Suite 300, Cincinnati, OH, 45242, USA |
| 66025 | Michigan Head Pain and Neurological Institute | Schulman Associates IRB | 4445 Lake Forest Drive, Suite 300, Cincinnati, OH, 45242, USA |
| 66028 | Thomas Jefferson University | Thomas Jefferson University IRB | 1020 Locust Street, Suite M‐34, Philadelphia, PA, 19107, USA |
| 66029 | Premiere Research Institute | Schulman Associates IRB | 4445 Lake Forest Drive, Suite 300, Cincinnati, OH, 45242, USA |
| 66033 | NeuroTrials Research | Schulman Associates IRB | 4445 Lake Forest Drive, Suite 300, Cincinnati, OH, 45242, USA |
| 66034 | Cleveland Clinic | Cleveland Clinic Institutional Review Board | 9500 Euclid Avenue, Mail Code OS 1, Cleveland, OH, 44195, USA |
| 66038 | Neurological Research Institute | Schulman Associates IRB | 4445 Lake Forest Drive, Suite 300, Cincinnati, OH, 45242, USA |
| 66039 | MedVadis Research | Schulman Associates IRB | 4445 Lake Forest Drive, Suite 300, Cincinnati, OH, 45242, USA |
| 66041 | Josephson Wallack Munshower Neurology | Schulman Associates IRB | 4445 Lake Forest Drive, Suite 300, Cincinnati, OH, 45242, USA |
| 66043 | Palm Beach Neurological Center | Schulman Associates IRB | 4445 Lake Forest Drive, Suite 300, Cincinnati, OH, 45242, USA |
| 66048 | Medical College of Wisconsin | Medical College of Wisconsin Froedtert Hospital Institutional Review Board | 8701 Watertown Plank Road, HRC‐MACC FUND 3040, Milwaukee, WI, 53226, USA |
| 66051 | Schuster Medical Research Institute | Schulman Associates IRB | 4445 Lake Forest Drive, Suite 300, Cincinnati, OH, 45242, USA |
| 66052 | FutureSearch Clinical Trials, LP | Schulman Associates IRB | 4445 Lake Forest Drive, Suite 300, Cincinnati, OH, 45242, USA |
| 66053 | Dent Neurologic Institute | Schulman Associates IRB | 4445 Lake Forest Drive, Suite 300, Cincinnati, OH, 45242, USA |
| 66054 | Clinical NeuroScience Solutions | Schulman Associates IRB | 4445 Lake Forest Drive, Suite 300, Cincinnati, OH, 45242, USA |
| 66055 | Clinical Neuroscience Solutions | Schulman Associates IRB | 4445 Lake Forest Drive, Suite 300, Cincinnati, OH, 45242, USA |

### **Phase 3 episodic migraine studies**

### **STRIVE study (ClinicalTrials.gov identifier: NCT02456740)**

| **Site no.** | **Site name** | **Ethics Committee or  Institutional Review Board** | **City, State/Province, Postal Code Country** |
| --- | --- | --- | --- |
| 12001 | Medizinische Universitaet  Innsbruck | Ethikkommission des Landes Oberoesterreich | Wagner‐Jauregg Weg 15, Linz, 4020, Austria |
| 12001 | Medizinische Universitaet  Innsbruck | Ethikkommission des Landes Oberoesterreich | Wagner‐Jauregg Weg 15, Linz, 4020, Austria |
| 12004 | Kepler Universitaetsklinikum GmbH | Ethikkommission des Landes Oberoesterreich | Wagner‐Jauregg Weg 15, Linz, 4020, Austria |
| 12004 | Kepler Universitaetsklinikum GmbH | Ethikkommission des Landes Oberoesterreich | Wagner‐Jauregg Weg 15, Linz, 4020, Austria |
| 12005 | Medizinische Universitaet  Wien | Ethikkommission des Landes Oberoesterreich | Wagner‐Jauregg Weg 15, Linz, 4020, Austria |
| 12005 | Medizinische Universitaet  Wien | Ethikkommission des Landes Oberoesterreich | Wagner‐Jauregg Weg 15, Linz, 4020, Austria |
| 12007 | Krankenhaus Hietzing mit Neurologischem Zentrum Rosenhuegel | Ethikkommission des Landes Oberoesterreich | Wagner‐Jauregg Weg 15, Linz, 4020, Austria |
| 12007 | Krankenhaus Hietzing mit Neurologischem Zentrum Rosenhuegel | Ethikkommission des Landes Oberoesterreich | Wagner‐Jauregg Weg 15, Linz, 4020, Austria |
| 13001 | Universitair Ziekenhuis  Gent | Universitair Ziekenhuis Gent – Ethisch Comite | De Pintelaan 185, De Pintepark 2, Verdiep 2, Campus Univeristair Ziekenhuis Gent, Gent, 9000, Belgium |
| 13002 | Centre Hospitalier Regional de la Citadelle | Comite d'Ethique Centre Hospitalier Regional de la Citadelle | Boulevard du 12eme de Ligne 1, Liege, 4000, Belgium |
| 13002 | Centre Hospitalier Regional de la Citadelle | Universitair Ziekenhuis Gent – Ethisch Comite | De Pintelaan 185, De Pintepark 2, Verdiep 2, Campus Univeristair Ziekenhuis Gent, Gent, 9000, Belgium |
| 13003 | Jessa Ziekenhuis −  Campus Virga Jesse | Universitair Ziekenhuis Gent – Ethisch Comite | De Pintelaan 185, De Pintepark 2, Verdiep 2, Campus Univeristair Ziekenhuis Gent, Gent, 9000, Belgium |
| 13003 | Jessa Ziekenhuis –  Campus Virga Jesse | Jessa Ziekenhuis – Ethische toetsingscommissie | Stadsomvaart 11, Hasselt, 3500, Belgium |
| 13004 | Centre Hospitalier Universitaire de Charleroi Hopital Civil Marie Curie | Comite d Ethique Centre Hospitalier Universitaire de Charleroi | Rue de Gozee 706, Montigny‐le‐Tilleul, 6110, Belgium |
| 13004 | Centre Hospitalier Universitaire de Hopital Civil Marie Curie | Universitair Ziekenhuis Gent – Ethisch Comite | De Pintelaan 185, De Pintepark 2, Verdiep 2, Campus Univeristair Ziekenhuis Gent, Gent, 9000, Belgium |
| 13005 | Universitair Ziekenhuis  Brussel | Universitair Ziekenhuis Gent – Ethisch Comite | De Pintelaan 185, De Pintepark 2, Verdiep 2, Campus Univeristair Ziekenhuis Gent, Gent, 9000, Belgium |
| 13005 | Universitair Ziekenhuis  Brussel | Universitair Ziekenhuis Brussel –  Commissie Medische Ethiek | Laarbeeklaan 101, Brussel, 1090, Belgium |
| 16001 | Ocean West Research Clinic Incorporated | Research Review Board, Inc. | 13085 Yonge Street, Unit 19, Suite 203, Richmond Hill, ON, L4E 0K2, Canada |
| 16001 | Ocean West Research Clinic Incorporated | Ocean West Research Clinic | 15850 26th Avenue, Suite 101, Surrey, BC, V3Z 2N6, Canada |
| 16003 | The Shapero Markham Headache and Pain Treatment Centre | Research Review Board, Inc. | 13085 Yonge Street, Unit 19, Suite 203, Richmond Hill, ON, L4E 0K2, Canada |
| 16004 | Ottawa Headache Centre Research Incorporated | Ottawa Headache Research Centre | 1 Centrepointe Drive, Suite 407, Ottawa, ON, K2G 6E2, Canada |
| 16004 | Ottawa Headache Centre  Research Incorporated | Research Review Board, Inc. | 13085 Yonge Street, Unit 19, Suite 203, Richmond Hill, ON, L4E 0K2, Canada |
| 16005 | Manna Research Incorporated | Research Review Board, Inc. | 13085 Yonge Street, Unit 19, Suite 203, Richmond Hill, ON, L4E 0K2, Canada |
| 21001 | Dado Medical sro | Eticka komise pri IKEM a TN | Videnska 800, Praha 4, 140 59, Czech Republic |
| 21002 | Thomayerova nemocnice | Eticka komise pri IKEM a TN | Videnska 800, Praha 4, 140 59, Czech Republic |
| 21003 | Fakultni nemocnice u svate Anny v Brne | Eticka komise Fakultni Nemocnice u sv Anny v Brne | Pekarska 53, Brno, 656 91, Czech Republic |
| 21005 | Mudr Stanislav Bartek sro | Eticka komise pri IKEM a TN | Videnska 800, Praha 4, 140 59, Czech Republic |
| 21006 | Poliklinika Chocen, Neurohk sro | Eticka komise pri IKEM a TN | Videnska 800, Praha 4, 140 59, Czech Republic |
| 21007 | Center for Clinical and Basic Research Pardubice, as | Eticka komise pri IKEM a TN | Videnska 800, Praha 4, 140 59, Czech Republic |
| 24001 | Helsingin Paansarkykeskus Aava | HUS Ethics Committee (HUS Tutkimuseettiset toimikunnat) | Tukholmankatu 8 C, Biomedicum Helsinki 2C, PL 705, HUS, 00029, Finland |
| 24003 | Terveystalo Oulu, Diapolis | HUS Ethics Committee (HUS Tutkimuseettiset toimikunnat) | Tukholmankatu 8 C, Biomedicum Helsinki 2C, PL 705, HUS, 00029, Finland |
| 24005 | Terveystalo Pulssi | HUS Ethics Committee (HUS Tutkimuseettiset toimikunnat) | Tukholmankatu 8 C, Biomedicum Helsinki 2C, PL 705, HUS, 00029, Finland |
| 24008 | Terveystalo Kamppi | HUS Ethics Committee (HUS Tutkimuseettiset toimikunnat) | Tukholmankatu 8 C, Biomedicum Helsinki 2C, PL 705, HUS, 00029, Finland |
| 24009 | Terveystalo Kuopio | HUS Ethics Committee (HUS Tutkimuseettiset toimikunnat) | Tukholmankatu 8 C, Biomedicum Helsinki 2C, PL 705, HUS, 00029, Finland |
| 48002 | Szpital Kliniczny im H Swiecickiego Uniwersytetu Medycznego im K Marcinkowskiego w Poznaniu | Komisja Bioetyczna przy Okregowej Izbie Lekarskiej w Lodzi | ulica Czerwona 3, Lodz,  93‐005, Poland |
| 48003 | Gabinet Lekarski Jacek Rozniecki | Komisja Bioetyczna przy Okregowej Izbie Lekarskiej w Lodzi | ulica Czerwona 3, Lodz,  93‐005, Poland |
| 48004 | Lubelskie Centrum Diagnostyczne | Komisja Bioetyczna przy Okregowej Izbie Lekarskiej w Lodzi | ulica Czerwona 3, Lodz,  93‐005, Poland |
| 48005 | Indywidualna Praktyka Lekarska dr hab Konrad Rejdak | Komisja Bioetyczna przy Okregowej Izbie Lekarskiej w Lodzi | ulica Czerwona 3, Lodz,  93‐005, Poland |
| 48006 | Samodzielny Publiczny Centralny Szpital Kliniczny | Komisja Bioetyczna przy Okregowej Izbie Lekarskiej w Lodzi | ulica Czerwona 3, Lodz,  93‐005, Poland |
| 48007 | Krakowska Akademia Neurologii Spzoo | Komisja Bioetyczna przy Okregowej Izbie Lekarskiej w Lodzi | ulica Czerwona 3, Lodz,  93‐005, Poland |
| 54001 | Univerzitna nemocnica Bratislava − Nemocnica akademika Ladislava Derera | Eticka komisia Univerzitnej nemocnice Bratislava – Nemocnica akademika Ladislava Derera | Limbova 5, Bratislava, 833 05, Slovakia |
| 54001 | Univerzitna nemocnica Bratislava – Nemocnica akademika Ladislava Derera | Eticka komisia Univerzitnej nemocnice Bratislava – Nemocnica akademika Ladislava Derera | Limbova 5, Bratislava, 833 05, Slovakia |
| 54002 | Vseobecna nemocnica s poliklinikou Lucenec no | Eticka komisia pri Vseobecnej nemocnici s poliklinikou Lucenec no | Namestie republiky 15, Lucenec, 984 39, Slovakia |
| 54002 | Vseobecna nemocnica s poliklinikou Lucenec no | Eticka komisia Univerzitnej nemocnice Bratislava ‐ Nemocnica akademika Ladislava Derera | Limbova 5, Bratislava, 833 05, Slovakia |
| 54002 | Vseobecna nemocnica s poliklinikou Lucenec no | Eticka komisia Univerzitnej nemocnice Bratislava ‐ Nemocnica akademika Ladislava Derera | Limbova 5, Bratislava, 833 05, Slovakia |
| 54003 | Forlife no Vseobecna nemocnica Komarno | Eticka komisia Univerzitnej nemocnice Bratislava − Nemocnica akademika Ladislava Derera | Limbova 5, Bratislava, 833 05, Slovakia |
| 54003 | Forlife no Vseobecna nemocnica Komarno | Eticka komisia pri Forlife no, Vseobecnej nemocnici Komarno | Medercska 39, Komarno,  945 75, Slovakia |
| 54003 | Forlife no Vseobecna nemocnica Komarno | Eticka komisia Univerzitnej nemocnice Bratislava − Nemocnica akademika Ladislava Derera | Limbova 5, Bratislava, 833 05, Slovakia |
| 63001 | Uludag Universitesi | Uludag University Faculty Of Medicine Ethical Committee of Clinical Research | Uludag Universitesi Tip Fakultesi Dekanligi, Nilufer, Bursa, 16059, Turkey |
| 65001 | John Radcliffe Hospital | East of England – Cambridge South REC | The Old Chapel, Royal Standard Place, Nottingham, NG1 6FS, United Kingdom |
| 65004 | Queen Elizabeth University Hospital | East of England – Cambridge South REC | The Old Chapel, Royal Standard Place, Nottingham, NG1 6FS, United Kingdom |
| 65005 | The Walton Centre NHS Foundation Trust | East of England – Cambridge South REC | The Old Chapel, Royal Standard Place, Nottingham, NG1 6FS, United Kingdom |
| 65008 | Kings College Hospital | East of England – Cambridge South REC | The Old Chapel, Royal Standard Place, Nottingham, NG1 6FS, United Kingdom |
| 65009 | Medinova Limited | East of England – Cambridge South REC | The Old Chapel, Royal Standard Place, Nottingham, NG1 6FS, United Kingdom |
| 65010 | Medinova Limited | East of England – Cambridge South REC | The Old Chapel, Royal Standard Place, Nottingham, NG1 6FS, United Kingdom |
| 65018 | Royal Stoke University Hospital | East of England – Cambridge South REC | The Old Chapel, Royal Standard Place, Nottingham, NG1 6FS, United Kingdom |
| 65019 | Medinova Limited | East of England – Cambridge South REC | The Old Chapel, Royal Standard Place, Nottingham, NG1 6FS, United Kingdom |
| 66001 | Clinical Research Institute Inc. | Schulman Associates IRB | 4445 Lake Forest Drive, Suite 300, Cincinnati, OH, 45242, USA |
| 66002 | Mercy Health Research | Mercy Hospital St Louis Institutional Review Board | 621 South New Ballas Road, Suite 6002B, St. Louis, MO, 63141, USA |
| 66002 | Mercy Health Research | Mercy Hospital Saint Louis | 615 South New Ballas Road, St Louis, MO, 63141, USA |
| 66002 | Mercy Health Research | Schulman Associates IRB | 4445 Lake Forest Drive, Suite 300, Cincinnati, OH, 45242, USA |
| 66003 | Nashville Neuroscience Group | Schulman Associates IRB | 4445 Lake Forest Drive, Suite 300, Cincinnati, OH, 45242, USA |
| 66004 | Clinvest Research LLC | Schulman Associates IRB | 4445 Lake Forest Drive, Suite 300, Cincinnati, OH, 45242, USA |
| 66005 | Rochester Clinical Research Inc. | Schulman Associates IRB | 4445 Lake Forest Drive, Suite 300, Cincinnati, OH, 45242, USA |
| 66006 | Heartland Research Associates LLC | Schulman Associates IRB | 4445 Lake Forest Drive, Suite 300, Cincinnati, OH, 45242, USA |
| 66007 | Encompass Clinical Research | Schulman Associates IRB | 4445 Lake Forest Drive, Suite 300, Cincinnati, OH, 45242, USA |
| 66008 | New England Regional Headache Center Inc. | Schulman Associates IRB | 4445 Lake Forest Drive, Suite 300, Cincinnati, OH, 45242, USA |
| 66010 | Premiere Research Institute | Schulman Associates IRB | 4445 Lake Forest Drive, Suite 300, Cincinnati, OH, 45242, USA |
| 66011 | Ohio Clinical Research LLC | Schulman Associates IRB | 4445 Lake Forest Drive, Suite 300, Cincinnati, OH, 45242, USA |
| 66012 | Charlottesville Medical Research Center | Schulman Associates IRB | 4445 Lake Forest Drive, Suite 300, Cincinnati, OH, 45242, USA |
| 66013 | Headache Wellness Center | Schulman Associates IRB | 4445 Lake Forest Drive, Suite 300, Cincinnati, OH, 45242, USA |
| 66014 | Rapid Medical Research Inc. | Schulman Associates IRB | 4445 Lake Forest Drive, Suite 300, Cincinnati, OH, 45242, USA |
| 66014 | Rapid Medical Research Inc. | Elite Research Network LLC | 501 Bramson Court, Suite 201, P.O. Box 2334, Mount Pleasant, SC, 29464, USA |
| 66017 | Otrimed Clinical Research | Schulman Associates IRB | 4445 Lake Forest Drive, Suite 300, Cincinnati, OH, 45242, USA |
| 66019 | Northwest Clinical Trials Inc. | Schulman Associates IRB | 4445 Lake Forest Drive, Suite 300, Cincinnati, OH, 45242, USA |
| 66020 | MediSphere Medical Research Center LLC | Schulman Associates IRB | 4445 Lake Forest Drive, Suite 300, Cincinnati, OH, 45242, USA |
| 66021 | Anaheim Clinical Trials | Schulman Associates IRB | 4445 Lake Forest Drive, Suite 300, Cincinnati, OH, 45242, USA |
| 66022 | Focus and Balance Research, LLC | Schulman Associates IRB | 4445 Lake Forest Drive, Suite 300, Cincinnati, OH, 45242, USA |
| 66023 | Clinical Neuroscience Solutions | Schulman Associates IRB | 4445 Lake Forest Drive, Suite 300, Cincinnati, OH, 45242, USA |
| 66024 | FutureSearch Clinical Trials, LP | Schulman Associates IRB | 4445 Lake Forest Drive, Suite 300, Cincinnati, OH, 45242, USA |
| 66025 | Advanced Clinical Research | Schulman Associates IRB | 4445 Lake Forest Drive, Suite 300, Cincinnati, OH, 45242, USA |
| 66026 | Suncoast Research Group LLC | Schulman Associates IRB | 4445 Lake Forest Drive, Suite 300, Cincinnati, OH, 45242, USA |
| 66028 | New England Institute for Clinical Research | Schulman Associates IRB | 4445 Lake Forest Drive, Suite 300, Cincinnati, OH, 45242, USA |
| 66029 | Bradenton Research Center | Schulman Associates IRB | 4445 Lake Forest Drive, Suite 300, Cincinnati, OH, 45242, USA |
| 66030 | Clinical Neuroscience Solutions | Schulman Associates IRB | 4445 Lake Forest Drive, Suite 300, Cincinnati, OH, 45242, USA |
| 66031 | Dent Neurologic Institute | Schulman Associates IRB | 4445 Lake Forest Drive, Suite 300, Cincinnati, OH, 45242, USA |
| 66032 | Carolina Women’s Research and Wellness Center | Schulman Associates IRB | 4445 Lake Forest Drive, Suite 300, Cincinnati, OH, 45242, USA |
| 66036 | NervePro | Schulman Associates IRB | 4445 Lake Forest Drive, Suite 300, Cincinnati, OH, 45242, USA |
| 66037 | Summit Research Network | Schulman Associates IRB | 4445 Lake Forest Drive, Suite 300, Cincinnati, OH, 45242, USA |
| 66039 | PANDA Neurology and Atlanta Headache Specialists | Schulman Associates IRB | 4445 Lake Forest Drive, Suite 300, Cincinnati, OH, 45242, USA |
| 66040 | Novex Clinical Research | Schulman Associates IRB | 4445 Lake Forest Drive, Suite 300, Cincinnati, OH, 45242, USA |
| 66041 | J Lewis Research | Schulman Associates IRB | 4445 Lake Forest Drive, Suite 300, Cincinnati, OH, 45242, USA |
| 66042 | Compass Research North | Schulman Associates IRB | 4445 Lake Forest Drive, Suite 300, Cincinnati, OH, 45242, USA |
| 66044 | Renstar Medical Research | Schulman Associates IRB | 4445 Lake Forest Drive, Suite 300, Cincinnati, OH, 45242, USA |
| 66045 | Thomas Jefferson University | Thomas Jefferson University IRB | 1020 Locust Street, Suite M‐34, Philadelphia, PA, 19107, USA |
| 66046 | Infinity Clinical Research | Schulman Associates IRB | 4445 Lake Forest Drive, Suite 300, Cincinnati, OH, 45242, USA |
| 66047 | Chase Medical Research LLC | Schulman Associates IRB | 4445 Lake Forest Drive, Suite 300, Cincinnati, OH, 45242, USA |
| 66048 | Arizona Research Center, Inc. | Schulman Associates IRB | 4445 Lake Forest Drive, Suite 300, Cincinnati, OH, 45242, USA |
| 66050 | Cleveland Clinic | Cleveland Clinic Institutional Review Board | 9500 Euclid Avenue, Mail Code OS 1, Cleveland, OH, 44195, USA |
| 66051 | Break Through Medical Research | Schulman Associates IRB | 4445 Lake Forest Drive, Suite 300, Cincinnati, OH, 45242, USA |
| 66052 | Alta California Medical Group | Schulman Associates IRB | 4445 Lake Forest Drive, Suite 300, Cincinnati, OH, 45242, USA |
| 66054 | Island Neurological Associates | Schulman Associates IRB | 4445 Lake Forest Drive, Suite 300, Cincinnati, OH, 45242, USA |
| 66055 | New Orleans Center For Clinical Research | Schulman Associates IRB | 4445 Lake Forest Drive, Suite 300, Cincinnati, OH, 45242, USA |
| 66056 | Clinical NeuroScience Solutions | Schulman Associates IRB | 4445 Lake Forest Drive, Suite 300, Cincinnati, OH, 45242, USA |
| 66057 | Schuster Medical Research Institute | Schulman Associates IRB | 4445 Lake Forest Drive, Suite 300, Cincinnati, OH, 45242, USA |
| 66059 | Protenium Clinical Research | Schulman Associates IRB | 4445 Lake Forest Drive, Suite 300, Cincinnati, OH, 45242, USA |
| 66060 | The Hartford Headache Center | Schulman Associates IRB | 4445 Lake Forest Drive, Suite 300, Cincinnati, OH, 45242, USA |
| 66061 | Partners in Clinical Research | Schulman Associates IRB | 4445 Lake Forest Drive, Suite 300, Cincinnati, OH, 45242, USA |
| 66062 | Desert Valley Research | Schulman Associates IRB | 4445 Lake Forest Drive, Suite 300, Cincinnati, OH, 45242, USA |
| 66063 | Pharmacology Research Institute | Schulman Associates IRB | 4445 Lake Forest Drive, Suite 300, Cincinnati, OH, 45242, USA |
| 66064 | Pharmacology Research Institute | Schulman Associates IRB | 4445 Lake Forest Drive, Suite 300, Cincinnati, OH, 45242, USA |
| 66064 | Pharmacology Research Institute | Pharmacology Research Institute | 6345 Balboa Boulevard, Building III, Suite 363, Encino, CA, 91316, USA |
| 66065 | Pharmacology Research Institute | Schulman Associates IRB | 4445 Lake Forest Drive, Suite 300, Cincinnati, OH, 45242, USA |
| 66066 | Anderson Clinical Research | Schulman Associates IRB | 4445 Lake Forest Drive, Suite 300, Cincinnati, OH, 45242, USA |
| 66067 | UnityPoint Health, Methodist Plaza Specialty Clinic | UnityPoint Health Des Moines | 700 East University, Suite West, Des Moines, IA, 50316, USA |
| 66067 | UnityPoint Health, Methodist Plaza Specialty Clinic | Schulman Associates IRB | 4445 Lake Forest Drive, Suite 300, Cincinnati, OH, 45242, USA |
| 66068 | Heartland Research Associates, LLC | Schulman Associates IRB | 4445 Lake Forest Drive, Suite 300, Cincinnati, OH, 45242, USA |
| 66070 | The Center for Pharmaceutical Research | Schulman Associates IRB | 4445 Lake Forest Drive, Suite 300, Cincinnati, OH, 45242, USA |
| 66072 | Alpine Clinical Research Center | Schulman Associates IRB | 4445 Lake Forest Drive, Suite 300, Cincinnati, OH, 45242, USA |

### **ARISE study (ClinicalTrials.gov identifier: NCT02483585)**

| **Site No.** | **Site Name** | **Ethics Committee or  Institutional Review Board** | **City, State/Province, Postal Code Country** |
| --- | --- | --- | --- |
| 22006 | Center for Clinical and Basic Research Aalborg | Den Videnskabsetiske Komité for Region Hovedstaden | Regionsgården Kongens Vænge 2, Hillerød, 3400, Denmark |
| 22001 | Glostrup Hospital | Den Videnskabsetiske Komité for Region Hovedstaden | Regionsgården Kongens Vænge 2, Hillerød, 3400, Denmark |
| 22004 | Centre for Clinical and Basic Research Ballerup | Den Videnskabsetiske Komité for Region Hovedstaden | Regionsgården Kongens Vænge 2, Hillerød, 3400, Denmark |
| 22005 | Center for Clinical and Basic Research Vejle | Den Videnskabsetiske Komité for Region Hovedstaden | Regionsgården Kongens Vænge 2, Hillerød, 3400, Denmark |
| 25001 | Centre Hospitalier Universitaire de Nice ‐ Hopital de Cimiez | Comite de Protection des Personnes Sud Mediterranee V Hopital de Cimiez | 4 avenue Reine Victoria, CS 91179,  Nice Cedex 1, 06003, France |
| 25002 | Centre Hospitalier Annecy Genevois | Comite de Protection des Personnes Sud Mediterranee V Hopital de Cimiez | 4 avenue Reine Victoria, CS 91179,  Nice Cedex 1, 06003, France |
| 25005 | Hopital Lariboisiere | Comite de Protection des Personnes Sud Mediterranee V Hopital de Cimiez | 4 avenue Reine Victoria, CS 91179,  Nice Cedex 1, 06003, France |
| 25007 | Groupe hospitalier Paris Saint Joseph | Comite de Protection des Personnes Sud Mediterranee V Hopital de Cimiez | 4 avenue Reine Victoria, CS 91179,  Nice Cedex 1, 06003, France |
| 27001 | Navy Hospital of Athens | National Ethics Committee | 284, Mesogion Avenue, Cholargos, Athens, 15562, Greece |
| 27002 | Mediterraneo Hospital | National Ethics Committee | 284, Mesogion Avenue, Cholargos, Athens, 15562, Greece |
| 27003 | 401 General Military Hospital of Athens | National Ethics Committee | 284, Mesogion Avenue, Cholargos, Athens, 15562, Greece |
| 27005 | Euromedica General Clinic  of Thessaloniki | National Ethics Committee | 284, Mesogion Avenue, Cholargos, Athens, 15562, Greece |
| 49001 | Hospital da Luz, SA | Comissao de Etica para a Investigacao Clinica (CEIC) | Avenida do Brasil, 53 − Pavilhao, 17‐A, Parque de Saude de Lisboa, Lisboa, 1749‐004, Portugal |
| 49003 | Centro Hospitalar de Lisboa Norte, EPE − Hospital de Santa Maria | Comissao de Etica para a Investigacao Clinica (CEIC) | Avenida do Brasil, 53 − Pavilhao, 17‐A, Parque de Saude de Lisboa, Lisboa, 1749‐004, Portugal |
| 49004 | Hospital Professor Doutor Fernando Fonseca, EPE | Comissao de Etica para a Investigacao Clinica (CEIC) | Avenida do Brasil, 53 − Pavilhao, 17‐A, Parque de Saude de Lisboa, Lisboa, 1749‐004, Portugal |
| 49005 | Campus Neurologico Senior | Comissao de Etica para a Investigacao Clinica (CEIC) | Avenida do Brasil, 53 − Pavilhao, 17‐A, Parque de Saude de Lisboa, Lisboa, 1749‐004, Portugal |
| 51101 | SBEI of HPE First Saint‐ Petersburg State Medical University na Academic I P Pavlov of MoH of the RF | Local EC of First I.P. Pavlov State Medical University of Saint‐ Petersburg | 10 Rentgena street, Saint Petersburg, 197101, Russia |
| 51103 | SBEI of HPE Bashkir state medical university of MoH of the Russian Federation | Local Ethics Committee of SBEI of HPE Bashkir state medical university of MoH of RF | 3 Lenina street, Ufa, 450000, Russia |
| 51104 | LLC City neurological center Sibneyromed | Local Ethics committee of LLC City neurological center Sibneyromed | 37 Michurina street, Novosibirsk, 630091, Russia |
| 51105 | SBEI of HPE First Moscow state medical university na I M Sechenov of MoH of the Russia | LEC of SBEI of HPE I M  Sechenov First Moscow State Medical University of MoH and SD of RF | 8 Trubetskaya street, building 2,  Moscow, 119992, Russia |
| 51106 | Limited Liability Company University Clinic of headaches | Independent interdisciplinary Committee on ethical review of clinical trials | 51 Leningradskiy prospekt, Moscow, 125468, Russia |
| 58002 | Hospital Universitari Vall d Hebron | CEIC Hospital Universitari Vall d Hebron | Passeig de la Vall d Hebron 119‐129, Institut de Recerca Ed.  Maternoinfantil Pl 13, Barcelona, Cataluña, 08035, Spain |
| 58003 | Hospital Universitari i Politecnic La Fe | CEIC Hospital Universitari i Politecnic La Fe | Avenida Fernando Abril Martorell 106, Torre A Planta 7, Valencia, Comunidad Valenciana, 46026, Spain |
| 58003 | Hospital Universitari i Politecnic La Fe | CEIC Hospital Universitari Vall d Hebron | Passeig de la Vall d Hebron 119‐129, Institut de Recerca Ed.  Maternoinfantil Pl 13, Barcelona, Cataluña, 08035, Spain |
| 58005 | Hospital Clinico Universitario de Valencia | CEIC Hospital Clinico Universitario de Valencia, Fundacion de Investigacion | Avenida de Vicente Blasco Ibañez 17, Pabellon B, Planta 1, Valencia, Comunidad Valenciana, 46010, Spain |
| 58005 | Hospital Clinico Universitario de Valencia | CEIC Hospital Universitari Vall d Hebron | Passeig de la Vall d Hebron 119‐129, Institut de Recerca Ed.  Maternoinfantil Pl 13, Barcelona, Cataluña, 08035, Spain |
| 58006 | Hospital Clinico Universitario de Santiago | CEIC de Galicia − SERGAS | Rua de San Lazaro s/n, Santiago de Compostela, Galicia, 15781, Spain |
| 58006 | Hospital Clinico Universitario de Santiago | CEIC Hospital Universitari Vall d Hebron | Passeig de la Vall d Hebron 119‐129, Institut de Recerca Ed.  Maternoinfantil Pl 13, Barcelona, Cataluña, 08035, Spain |
| 58007 | Hospital Clínico Universitario de Valladolid | CEIC Area de Salud de Valladolid Este | Avenida Ramon y Cajal 3, Valladolid, Castilla León, 47005, Spain |
| 58007 | Hospital Clínico Universitario de Valladolid | CEIC Hospital Universitari Vall d Hebron | Passeig de la Vall d Hebron 119‐129, Institut de Recerca Ed.  Maternoinfantil Pl 13, Barcelona, Cataluña, 08035, Spain |
| 58008 | Hospital Clinico Universitario Lozano Blesa | CEIC de Aragon − CEICA | Avenida San Juan Bosco 13, Planta 1, Zaragoza, Aragón, 50009, Spain |
| 58008 | Hospital Clinico Universitario Lozano Blesa | CEIC Hospital Universitari Vall d Hebron | Passeig de la Vall d Hebron 119‐129, Institut de Recerca Ed.  Maternoinfantil Pl 13, Barcelona, Cataluña, 08035, Spain |
| 58010 | Hospital Universitario Marques de Valdecilla | CEIC de Cantabria | Avenida del Cardenal Herrera Oria s/n, Edificio IFIMAV Planta 3, Santander, Cantabria, 39011, Spain |
| 58010 | Hospital Universitario Marques de Valdecilla | CEIC Hospital Universitari Vall d Hebron | Passeig de la Vall d Hebron 119‐129, Institut de Recerca Ed.  Maternoinfantil Pl 13, Barcelona, Cataluña, 08035, Spain |
| 60001 | RehaClinic Bad Zurzach | Ethikkommission Nordwest − und Zentralschweiz (EKNZ) | Hebelstrasse 53, Basel, 4056, Switzerland |
| 60002 | Kantonsspital St Gallen | Ethikkommission Nordwest − und Zentralschweiz (EKNZ) | Hebelstrasse 53, Basel, 4056, Switzerland |
| 60004 | Ospedale Regionale di Lugano ‐ Civico | Ethikkommission Nordwest − und Zentralschweiz (EKNZ) | Hebelstrasse 53, Basel, 4056, Switzerland |
| 60006 | Kopfwehzentrum Hirslanden | Ethikkommission Nordwest − und Zentralschweiz (EKNZ) | Hebelstrasse 53, Basel, 4056, Switzerland |
| 60007 | Medizinisches Zentrum Biel | Ethikkommission Nordwest − und Zentralschweiz (EKNZ) | Hebelstrasse 53, Basel, 4056, Switzerland |
| 66002 | Tidewater Integrated Medical Research | Schulman Associates IRB | 4445 Lake Forest Drive, Suite 300, Cincinnati, OH, 45242, USA |
| 66003 | Josephson Wallack Munshower Neurology | Schulman Associates IRB | 4445 Lake Forest Drive, Suite 300, Cincinnati, OH, 45242, USA |
| 66004 | Texas Neurology, PA | Schulman Associates IRB | 4445 Lake Forest Drive, Suite 300, Cincinnati, OH, 45242, USA |
| 66005 | Neurological Research Institute | Schulman Associates IRB | 4445 Lake Forest Drive, Suite 300, Cincinnati, OH, 45242, USA |
| 66006 | Collaborative Neuroscience Network Inc. | Schulman Associates IRB | 4445 Lake Forest Drive, Suite 300, Cincinnati, OH, 45242, USA |
| 66007 | Westside Family Medical Center | Schulman Associates IRB | 4445 Lake Forest Drive, Suite 300, Cincinnati, OH, 45242, USA |
| 66008 | Michigan Head Pain and Neurological Institute | Schulman Associates IRB | 4445 Lake Forest Drive, Suite 300, Cincinnati, OH, 45242, USA |
| 66010 | Associated Neurologists of Southern Connecticut | Schulman Associates IRB | 4445 Lake Forest Drive, Suite 300, Cincinnati, OH, 45242, USA |
| 66011 | Ideas‐Vision‐Answers Research | Schulman Associates IRB | 4445 Lake Forest Drive, Suite 300, Cincinnati, OH, 45242, USA |
| 66013 | PMG Research of Winston Salem | Schulman Associates IRB | 4445 Lake Forest Drive, Suite 300, Cincinnati, OH, 45242, USA |
| 66014 | Albuquerque Clinical Trials Inc. | Schulman Associates IRB | 4445 Lake Forest Drive, Suite 300, Cincinnati, OH, 45242, USA |
| 66015 | ProScience Research Group | Schulman Associates IRB | 4445 Lake Forest Drive, Suite 300, Cincinnati, OH, 45242, USA |
| 66016 | Diablo Clinical Research | Schulman Associates IRB | 4445 Lake Forest Drive, Suite 300, Cincinnati, OH, 45242, USA |
| 66017 | Seattle Women’s Health Research Gynecology | Schulman Associates IRB | 4445 Lake Forest Drive, Suite 300, Cincinnati, OH, 45242, USA |
| 66018 | Artemis Institute for Clinical Research | Schulman Associates IRB | 4445 Lake Forest Drive, Suite 300, Cincinnati, OH, 45242, USA |
| 66020 | Clinical Research of Philadelphia LLC | Schulman Associates IRB | 4445 Lake Forest Drive, Suite 300, Cincinnati, OH, 45242, USA |
| 66021 | Princeton Medical Institute | Schulman Associates IRB | 4445 Lake Forest Drive, Suite 300, Cincinnati, OH, 45242, USA |
| 66022 | Oviedo Medical Research | Schulman Associates IRB | 4445 Lake Forest Drive, Suite 300, Cincinnati, OH, 45242, USA |
| 66023 | Northern California Research | Schulman Associates IRB | 4445 Lake Forest Drive, Suite 300, Cincinnati, OH, 45242, USA |
| 66024 | Achieve Clinical Research, LLC | Schulman Associates IRB | 4445 Lake Forest Drive, Suite 300, Cincinnati, OH, 45242, USA |
| 66025 | Midamerica Neuroscience Institute d/b/a Rowe Neurology Institute | Schulman Associates IRB | 4445 Lake Forest Drive, Suite 300, Cincinnati, OH, 45242, USA |
| 66028 | DermResearch Inc. | Schulman Associates IRB | 4445 Lake Forest Drive, Suite 300, Cincinnati, OH, 45242, USA |
| 66029 | Omega Medical Research | Schulman Associates IRB | 4445 Lake Forest Drive, Suite 300, Cincinnati, OH, 45242, USA |
| 66030 | Lynn Health Science Institute | Schulman Associates IRB | 4445 Lake Forest Drive, Suite 300, Cincinnati, OH, 45242, USA |
| 66032 | NeuroTrials Research | Schulman Associates IRB | 4445 Lake Forest Drive, Suite 300, Cincinnati, OH, 45242, USA |
| 66034 | ClinSearch LLC | Schulman Associates IRB | 4445 Lake Forest Drive, Suite 300, Cincinnati, OH, 45242, USA |
| 66034 | ClinSearch LLC | ClinSearch LLC | 6035 Shallowford Road, Suite 109, Chattanooga, TN, 37421, USA |
| 66036 | Colorado Springs Neurological Associates | Schulman Associates IRB | 4445 Lake Forest Drive, Suite 300, Cincinnati, OH, 45242, USA |
| 66038 | Compass Research | Schulman Associates IRB | 4445 Lake Forest Drive, Suite 300, Cincinnati, OH, 45242, USA |
| 66039 | Clinical Investigation Specialists Inc. | Schulman Associates IRB | 4445 Lake Forest Drive, Suite 300, Cincinnati, OH, 45242, USA |
| 66040 | Upstate Clinical Research Associates | Schulman Associates IRB | 4445 Lake Forest Drive, Suite 300, Cincinnati, OH, 45242, USA |
| 66042 | Asheville Neurology Specialists PA | Schulman Associates IRB | 4445 Lake Forest Drive, Suite 300, Cincinnati, OH, 45242, USA |
| 66043 | Pharmasite Research Inc. | Schulman Associates IRB | 4445 Lake Forest Drive, Suite 300, Cincinnati, OH, 45242, USA |
| 66045 | Coastal Carolina Research Center | Schulman Associates IRB | 4445 Lake Forest Drive, Suite 300, Cincinnati, OH, 45242, USA |
| 66047 | Infinity Clinical Research | Schulman Associates IRB | 4445 Lake Forest Drive, Suite 300, Cincinnati, OH, 45242, USA |
| 66048 | Boston Clinical Trials | Schulman Associates IRB | 4445 Lake Forest Drive, Suite 300, Cincinnati, OH, 45242, USA |
| 66049 | Jacksonville Center for Clinical Research | Schulman Associates IRB | 4445 Lake Forest Drive, Suite 300, Cincinnati, OH, 45242, USA |
| 66050 | J Lewis Research | Schulman Associates IRB | 4445 Lake Forest Drive, Suite 300, Cincinnati, OH, 45242, USA |
| 66051 | Elite Clinical Studies LLC | Schulman Associates IRB | 4445 Lake Forest Drive, Suite 300, Cincinnati, OH, 45242, USA |

### **LIBERTY study (ClinicalTrials.gov identifier: NCT03096834)**

| **Centre No.** | **Ethics Committee or  Institutional Review Board** | **Department / Organization** | **City, State/Province, Postal Code Country** |
| --- | --- | --- | --- |
| 1003 | Austin Health Human Research Ethics Committee | Office for Research | Heidelberg Victoria 3084 Australia |
| 1011 | Ethikkommission der Med.  Universität Wien | Ethikkommission | Vienna A-1090 Austria |
| 1013 | Ethikkommission der Med. Universität Wien | Ethikkommission | Vienna A-1090 Austria |
| 1021 | Commissie voor Medische Ethiek UZ Gent | Commissie Medische Ethiek | Gent 9000 Belgium |
| 1022 | Commissie voor Medische Ethiek UZ Gent | Commissie Medische Ethiek | Gent 9000 Belgium |
| 1023 | Commissie voor Medische Ethiek UZ Gent | Commissie Medische Ethiek | Gent 9000 Belgium |
| 1024 | Commissie voor Medische Ethiek UZ Gent | Commissie Medische Ethiek | Gent 9000 Belgium |
| 1031 | Etická komise IKEM a TN, Thomayerova nemocnice | Vídeňská 800 | Praha 4 140 59 Czech Republic |
| 1033 | Etická komise IKEM a TN, Thomayerova nemocnice | Vídeňská 800 | Praha 4 140 59 Czech Republic |
| 1034 | Etická komise IKEM a TN, Thomayerova nemocnice | Vídeňská 800 | Praha 4 140 59 Czech Republic |
| 1041 | De Videnskabsetiske Komiteer for Region Hovedstaden | Regionsgården, Center for sundhed, Kongens Vænge 2 | Hillerød 3400 Denmark |
| 1051 | Varsinais-Suomen eettinen toimikunta |  | Turku 20521 Finland |
| 1053 | Varsinais-Suomen eettinen toimikunta |  | Turku 20521 Finland |
| 1054 | Varsinais-Suomen eettinen toimikunta |  | Turku 20521 Finland |
| 1061 | Comite de protection des personnes Sud Mediterranee I |  | Marseille 13274 France |
| 1063 | Comite de protection des personnes Sud Mediterranee I |  | Marseille 13274 France |
| 1065 | Comite de protection des personnes Sud Mediterranee I |  | Marseille 13274 France |
| 1071 | Landesamt für Gesundheit und Soziales, Ethik-Kommission des Landes Berlin |  | Berlin 10707 Germany |
| 1072 | Ludwig-Maximilians-Universität München, Medizinische Fakultät Ethikkommission |  | München 80336 Germany |
| 1073 | Christian-Albrechts-Universität zu Kiel, Ethik-Kommission der Medizinischen Fakultät |  | Kiel 24105 Germany |
| 1074 | Ärztekammer Hamburg, Geschäftsstelle der Ethik-Kommission |  | Hamburg 22083 Germany |
| 1075 | Landesärztekammer Hessen, Ethik-Kommission |  | Frankfurt am Main 60488 Germany |
| 1076 | Landesamt für Gesundheit und Soziales, Ethik-Kommission des Landes Berlin |  | Berlin 10707 Germany |
| 1077 | Sächsische Landesärztekammer, Ethik-Kommission |  | Dresden 01099 Germany |
| 1078 | Universitätsklinikum Essen, Medizinische Fakultät der Universität Duisburg-Essen Ethik-Kommission |  | Essen 45147 Germany |
| 1079 | Ethik-Kommission an der Medizinischen Fakultät der Eberhard-Karls-Universität und am Universitätsklinikum Tübingen |  | Tübingen 72074 Germany |
| 1081 | Ethik-Kommission der Ärztekammer Westfalen-Lippe und der Westfälischen Wilhelms-Universität Münster |  | Münster 48147 Germany |
| 1082 | Ethik-Kommission der Ärztekammer Westfalen-Lippe und der Westfälischen Wilhelms-Universität Münster |  | Münster 48147 Germany |
| 1084 | Friedrich-Alexander-Universität Erlangen-Nürnberg, Medizinische Fakultät Ethik-Kommission |  | Erlangen 91054 Germany |
| 2001 | National Ethics Committee |  | Cholargos Attica 15562 Greece |
| 2003 | National Ethics Committee |  | Cholargos Attica 15562 Greece |
| 2004 | National Ethics Committee |  | Cholargos Attica 15562 Greece |
| 2011 | Comitato Etico dell'Università Sapienza | Policlinico Universitario Umberto I | Roma 00161 Italy |
| 2012 | Comitato Etico Regione Toscana Area Vasta Centro | Azienda Ospedaliero Universitaria Careggi di Firenze | Firenze 50134 Italy |
| 2014 | Comitato Etico Palermo | AOU Policlinico P. Giaccone di Palermo | Palermo 90127 Italy |
| 2015 | Comitato Etico Seconda Università degli Studi di Napoli - Università degli Studi della Campania L. Vanvitelli | AOU SUN-AORN Ospedale dei Colli | Napoli 80138 Italy |
| 2016 | Comitato Etico Regione Lombardia - Sezione della Fondazione IRCCS | Istituto Neurologico Carlo Besta | Milano 20133 Italy |
| 2017 | Comitato Etico di Area Vasta Emilia Centro della Regione Emilia-Romagna CE AVEC | AOU di Bologna, Policlinico S.Orsola-Malpighi | Bologna 40138 Italy |
| 2028 | METC LUCM | Commissie Medische Ethiek | Leiden N/A 2300 PC the Netherlands |
| 2029 | METC LUCM | Commissie Medische Ethiek | Leiden N/A 2300 PC the Netherlands |
| 2030 | METC LUCM | Commissie Medische Ethiek | Leiden N/A 2300 PC the Netherlands |
| 2041 | Regionale komiteer for medisinsk og helsefaglig forskningsetikk | REK sor-ost B | Oslo 0484 Norway |
| 2043 | Regionale komiteer for medisinsk og helsefaglig forskningsetikk | REK sor-ost B | Oslo 0484 Norway |
| 2051 | CEIC Grupo Hospitalario Quirón en Barcelona | Centro Médico Teknon | Barcelona Barcelona 08022 Spain |
| 2052 | CEIC Grupo Hospitalario Quirón en Barcelona | Centro Médico Teknon | Barcelona Barcelona 08022 Spain |
| 2053 | CEIC Grupo Hospitalario Quirón en Barcelona | Centro Médico Teknon | Barcelona Barcelona 08022 Spain |
| 2055 | CEIC Grupo Hospitalario Quirón en Barcelona | Centro Médico Teknon | Barcelona Barcelona 08022 Spain |
| 2056 | CEIC Grupo Hospitalario Quirón en Barcelona | Centro Médico Teknon | Barcelona Barcelona 08022 Spain |
| 2057 | CEIC Grupo Hospitalario Quirón en Barcelona | Centro Médico Teknon | Barcelona Barcelona 08022 Spain |
| 2067 | Etikprövningsmyngdigheten |  | Uppsala 750 02 Sweden |
| 2069 | Etikprövningsmyngdigheten |  | Uppsala 750 02 Sweden |
| 2070 | Etikprövningsmyngdigheten |  | Uppsala 750 02 Sweden |
| 2072 | Etikprövningsmyngdigheten |  | Uppsala 750 02 Sweden |
| 2073 | Etikprövningsmyngdigheten |  | Uppsala 750 02 Sweden |
| 2081 | Kantonale Ethikkommission Zürich |  | Zürich 8090 Switzerland |
| 2082 | Kantonale Ethikkommission Zürich |  | Zürich 8090 Switzerland |
| 2083 | Kantonale Ethikkommission Zürich |  | Zürich 8090 Switzerland |
| 2091 | East Midlands − Leicester Central Research Ethics Committee | The Old Chapel, Royal Standard Place | Nottingham NG1 6FS United Kingdom |
| 2092 | East Midlands − Leicester Central Research Ethics Committee | The Old Chapel, Royal Standard Place | Nottingham NG1 6FS United Kingdom |
| 2094 | East Midlands − Leicester Central Research Ethics Committee | The Old Chapel, Royal Standard Place | Nottingham NG1 6FS United Kingdom |

### **EMPOwER study (ClinicalTrials.gov identifier: NCT03333109)**

| **Centre No.** | **Ethics Committee or Institutional Review Board** | **Department / Organization** | **City, State/Province, Postal Code Country** |
| --- | --- | --- | --- |
| 1005 | Comité Independiente de Ética Para Ensayos En Farmacologia Clinica | Fundación De Estudios Farmacologia y de Medicamentos FEFyM | Buenos Aires, C1027AAP, Argentina |
| 1006 | Comité Independiente de Ética Para Ensayos En Farmacologia Clinica | Fundación De Estudios Farmacologia y de Medicamentos FEFyM | Buenos Aires, C1027AAP, Argentina |
| 1007 | Comité de Etica de Protocolos de Investigación del Hospital Italiano CABA – CEPI |  | Ciudad Autonoma de Buenos Aires, Buenos Aires, 1181, Argentina |
| 1008 | Comite de Etica del Centro de Osteopatias Medicas | Buenos Aires | Caba, Buenos Aires, C1128AAF, Argentina |
| 1009 | Comite Institucional de Etica de investigacion en salud del Sanatorio Allende |  | Cordoba, X5000, Argentina |
| 1010 | CEIB − Comité de Etica en Investigaciones Biomedicas | Instituto Fleni | Buenos Aires, C1428AQK, Argentina |
| 1011 | CINME |  | Ciudad Autónoma de Buenos Aires, Buenos Aires, C1056ABJ, Argentina |
| 1301 | Institutional Ethics Committee, Yashoda Hospitals | Yashoda Academy of Medical Education and Research | Hyderabad, Andhra Pradesh, 500003, India |
| 1302 | Independent Human Ethics Committee, Health and Research Centre |  | Trivandrum, Kerala, 695011, India |
| 1303 | Institutional Ethics Committee |  | Mumbai, Maharashtra, 400008, India |
| 1304 | Ethical Review Board | MS Ramaiah Medical College &Teaching Hospital | Bangalore, Karnataka, 560054, India |
| 1305 | Bhakti Vedanta Hospital Ethics Committee |  | Mumbai, Maharashtra, 401107, India |
| 1306 | Institutional Ethics Committee |  | Pune, Maharashtra, 411004, India |
| 1307 | Institutional Ethics Committee | Apollo Hospital International Limited | Ahmedabad, Gujrat, 382428, India |
| 1309 | Institutional Ethics Committee | Ethics Committee | Chennai, Tamilnadu, 600100, India |
| 1311 | Getwell Institution Ethics Committee |  | Nagpur, Maharashtra, 6632200, India |
| 1312 | Institutional Ethics Committee, |  | Mumbai, Maharashtra, 400016, India |
| 1313 | The Ethics Committee of Sri Aurobindo Seva Kendra | Sri Aurobindo Seva Kendra | Kolkata, West Bengal, 700068, India |
| 1314 | Drug trial Ethics Committee | Dayanand Medical College | Ludhiana, Punjab, 141001, India |
| 1315 | Institutional Ethics Committee | Sir Ganga Ram Hospital | New Delhi, Delhi, 110060, India |
| 1317 | Institutional Ethics Committee | Shri Guru Ram Rai Institute of Medical & Health Sciences | Dehradun, Uttrakhand, 248001, India |
| 1318 | Institutional Ethics Committee | Aster Medcity | Kochi, Kerala, 682027, India |
| 1321 | Vijan Hospital Ethics Committee | Vijan Cardiac & Critical Care Centre, Dr. Vijan Hospital Marg | Nashik, Maharashtra, 422005, India |
| 1322 | Institutional Ethics Committee | Baby Memorial Hospital | Kozhikode, Kerala, 673004, India |
| 1323 | Institutional Ethics Committee | AMRITA Institute of Medical Sciences And Research Center | Kochi, Kerala, 682041, India |
| 1324 | Institutional Ethics committee | Maulana Azad Medical College and associated Hospitals | New Delhi, 110006, India |
| 1325 | Institute Ethics Committee | All India Institute of Medical Sciences | New Delhi, 110029, India |
| 1326 | Institutional Ethics Committee | SGPGI Bioethics Cell, SGPGI, Raebareli Road | Lucknow, Uttar Pradesh, 2260147, India |
| 1327 | Institutional Ethics Committee | Kle Academy Of Higher Education & Research | Belagavi, Karnataka, 590010, India |
| 1328 | Institutional Ethics Committee - Mysore Medical College & research institute and Associated Hospitals |  | Mysore, Karnataka, 570021, India |
| 1329 | Institutional Ethics Committee | BGS Global Hospital | Bangalore, Karnataka, 560060, India |
| 1330 | Ethics committee | Lalitha Superspecialities Hospital Pvt. Ltd | Guntur, Andhra Pradesh, 522001, India |
| 1331 | Magna Care Ethics Committee, Nashik | Neurology | Nashik, Maharashtra, 422005, India |
| 1333 | Institutional Ethics Committee | Institute Of Neurosciences Kolkata | Kolkata, West Bengal, 700017, India |
| 1071 | Eulji General Hospital Institutional Review Board |  | Seoul, 01830, Republic of Korea, |
| 1073 | Seoul National University Hospital Institutional Review Board |  | Seoul, 03080, Republic of Korea, |
| 1074 | Korea University Guro Hospital Institutional Review Board |  | Seoul, 08308, Republic of Korea, |
| 1075 | Hallym University Dongtan Sacred Heart Hospital Institutional Review Board |  | Hwaweong-si, Gyeonggido, 18450, Republic of Korea, |
| 1076 | The Catholic University of Korea Uijeongbu St. Mary's Hospital Institutional Review Board |  | Uijeongbu, Gyeonggi do, 11765, Republic of Korea, |
| 1077 | Chungnam National University Hospital Institutional Review Board |  | Daejeon, 35015, Republic of Korea, |
| 1078 | Inha University Hospital Institutional Review Board |  | Incheon, 22332, Republic of Korea, |
| 1079 | Chonnam National University Hospital Institutional Review Board |  | Gwangju, 61469, Republic of Korea, |
| 1080 | Kangbuk Samsung Hospital Institutional Review Board |  | Seoul, 03181, Republic of Korea, |
| 1081 | Inje University Pusan Paik Hospital Institutional Review Board |  | Busan, 47392, Republic of Korea, |
| 1082 | Samsung Medical Center Institutional Review Board |  | Seoul, 06351, Republic of Korea, |
| 1083 | Yonsei University Severance Hospital Institutional Review Board |  | Seoul, 03722, Republic of Korea, |
| 1141 | Institutional Review Board, Rafik Hariri University Hospital |  | Beirut, Lebanon |
| 1142 | Institutional Review Board American University of Beirut | Biomedical | Beirut, 1107 2020, Lebanon |
| 1143 | Institutional review board Bellevue Medical Center | Medical | Mansourieh, Beirut, 295, Lebanon |
| 1144 | AWH Institutional Review Board | Institutional Review Board | El Chouf, Lebanon |
| 1145 | Institutional Review Board of Makassed General Hospital |  | Beirut, 6301, Lebanon |
| 1146 | Institutional Review Board, Rafik Hariri University Hospital |  | Beirut, Lebanon |
| 1147 | Institutional Review Board at Saint Georges Hospital University Medical Faculty |  | Beirut, 166378, Lebanon |
| 1101 | Medical Ethics Committee | University Malaya Medical Centre | Kuala Lumpur, 59100, Malaysia |
| 1102 | Medical Research & Ethics Committee | Ministry of Health Malaysia c/o Institute for Health Management | Kuala Lumpur, Wilayah Persekutuan, 59000, Malaysia |
| 1103 | Medical Research & Ethics Committee | Ministry of Health Malaysia c/o Institute for Health Management | Kuala Lumpur, Wilayah Persekutuan, 59000, Malaysia |
| 1104 | Medical Research & Ethics Committee | Ministry of Health Malaysia c/o Institute for Health Management | Kuala Lumpur, Wilayah Persekutuan, 59000, Malaysia |
| 1105 | Medical Research & Ethics Committee | Ministry of Health Malaysia c/o Institute for Health Management | Kuala Lumpur, Wilayah Persekutuan, 59000, Malaysia |
| 1121 | Comite de Etica en Investigacion de la Unidad de Investigacipon en Salud de Chihuahua |  | Chihuahua, 31203, Mexico |
| 1122 | Unidad Clínica de Bioequivalencia S. de R.L. de C.V. |  | Guadalajara, Jalisco, 44190, Mexico |
| 1123 | Instituto Jaliscience de invstigacion clinica SA de CV |  | Guadalajara, Jalisco, 4410, Mexico |
| 1125 | Hospital la Misión S.A. de C.V. |  | Monterrey, Nuevo León, 64718, Mexico |
| 1211 | St. Luke's Medical Center | Institutional Ethics Review Board | Quezon City, Metro Manila, 1102, Philippines |
| 1212 | St. Luke's Medical Center | Institutional Ethics Review Board | Quezon City, Metro Manila, 1102, Philippines |
| 1213 | Philippine General Hospital | University of the Philippines Manila Research Ethics Board | Manila, Metro Manila, 1000, Philippines |
| 1214 | The Medical City | Institutional Review Board | Pasig City, Metro Manila, 1605, Philippines |
| 1151 | SingHealth Centralized Institutional Review Board | Domain A | Singapore, 169611, |
| 1152 | SingHealth Centralized Institutional Review Board | Domain A | Singapore, 169611, |
| 1161 | Institutional Review Board | Taipei Veterans General Hospital | Taipei, 11217, Taiwan |
| 1162 | Mackay Memorial Hospital Institutional Review Board | Mackay Memorial Hospital | Taipei, 10449, Taiwan |
| 1163 | Institutional Review Board | Chang Gung Medical Foundation | Taoyuan, 333, Taiwan |
| 1164 | Kuang Tien General Hospital Institutional Review Board | Kuang Tien General Hospital | TaiChung, 437, Taiwan |
| 1165 | Chia-Yi Christian Hospital Institutional Review Board | Chia-Yi Christian Hospital | Chia-Yi City, Taiwan, 60002, Taiwan |
| 1166 | Chi Mei Medical Center Institutional Review Board | Chi Mei Medical Center | Tainan City, 71004, Taiwan |
| 1167 | Chi Mei Medical Center Institutional Review Board | Chi Mei Medical Center | Tainan City, 71004, Taiwan |
| 1168 | China Medical University Hospital Institutional Review Board | China Medical University Hospital | Taichung City, 404, Taiwan |
| 1181 | Research Ethics Committee | Faculty of Medicine Chiang Mai University | Chiang Mai, Chiang Mai, 50200, Thailand |
| 1182 | The Institutional Review Board | Faculty of Medicine, Chulalongkorn University | Bangkok, 10330, Thailand |
| 1183 | The Institutional Review Board Royal Thai Army Medical Department | Phramongkutklao Hospital | Bangkok, 10400, Thailand |
| 1184 | Prasat Neurological Institute Research Center | Prasat Neurological Institute | Bangkok, 10400, Thailand |
| 1185 | The Khon Kaen University Ethics Committee for Human Research | Srinagarind Hospital | Khon Kaen, 40002, Thailand |
| 1201 | Ethics Committee of HCMC University Medical center |  | Ho Chi Minh City, 700000, Vietnam |
| 1202 | Ethics Committee of Bach Mai Hospital |  | Hanoi, 100000, Vietnam |
